# Clinical, mechanistic, biomarker, and therapeutic advances in *GBA1*-associated Parkinson’s disease

**DOI:** 10.1186/s40035-024-00437-6

**Published:** 2024-09-12

**Authors:** Xuxiang Zhang, Heng Wu, Beisha Tang, Jifeng Guo

**Affiliations:** 1 Department of Neurology, Xiangya Hospital, Central South University, Changsha, 410008 China; 2 Department of Neurology, Multi-Omics Research Center for Brain Disorders, The First Affiliated Hospital, University of South China, Hengyang, 421001 China; 3 Clinical Research Center for Immune-Related Encephalopathy of Hunan Province, Hengyang, 421001 China; 4 Key Laboratory of Hunan Province in Neurodegenerative Disorders, Central South University, Changsha, 410008 China; 5 Hunan International Scientific and Technological Cooperation Base of Neurodegenerative and Neurogenetic Diseases, Changsha, 410008 China; 6 Center for Medical Genetics and Hunan Key Laboratory of Medical Genetics, School of Life Sciences, Central South University, Changsha, 410008 China; 7 Engineering Research Center of Hunan Province in Cognitive Impairment Disorders, Central South University, Changsha, 410008 China; 8 National Clinical Research Center for Geriatric Disorders, Xiangya Hospital, Central South University, Changsha, 410008 China

**Keywords:** Gaucher’s disease, Parkinson’s disease, Glucocerebrosidase, Mechanisms, Biomarker, Therapy

## Abstract

Parkinson’s disease (PD) is the second most common neurodegenerative disease. The development of PD is closely linked to genetic and environmental factors, with *GBA1* variants being the most common genetic risk. Mutations in the *GBA1* gene lead to reduced activity of the coded enzyme, glucocerebrosidase, which mediates the development of PD by affecting lipid metabolism (especially sphingolipids), lysosomal autophagy, endoplasmic reticulum, as well as mitochondrial and other cellular functions. Clinically, PD with *GBA1* mutations (*GBA1*-PD) is characterized by particular features regarding the progression of symptom severity. On the therapeutic side, the discovery of the relationship between *GBA1* variants and PD offers an opportunity for targeted therapeutic interventions. In this review, we explore the genotypic and phenotypic correlations, etiologic mechanisms, biomarkers, and therapeutic approaches of *GBA1*-PD and summarize the current state of research and its challenges.

**Supplementary Information:**

The online version contains supplementary material available at 10.1186/s40035-024-00437-6.

## Introduction

Parkinson’s disease (PD) is one of the most common neurological disorders characterized by motor symptoms such as resting tremor, bradykinesia, rigidity and postural instability, often accompanied by a variety of non-motor symptoms. The pathology of PD is characterized by the loss of dopaminergic neurons and the formation of α-synuclein (α-syn)-containing proteinaceous aggregates, known as Lewy bodies (LBs) and Lewy neurites [1]. The etiology of PD is not fully understood and its incidence increases with age, with genetic and environmental factors playing important roles in its onset and progression. With the rapid development of genetic testing technology, important advances have been made in the genetic aspects of PD [2]. To date, mutations in more than 20 genes and more than 90 common genetic risk loci have been reported to be associated with PD [2–7]. The roles of these genes and variants in PD remain controversial and require further validation and functional studies. Understanding the biological changes induced by these genetic variants is both important and challenging. Despite the complexity, studies of PD-associated genes have focused on a number of common pathogenic pathways, including lysosomal dysfunction, impaired intracellular trafficking, inflammatory signaling, and mitochondrial dysfunction. These pathways have been linked to dopaminergic neuronal damage [8].

Previous research has established a clear correlation between genes regulating lysosomal function and PD [9]. Recent studies revealed that over 50% of individuals with PD possess one or more putative damaging variants among the lysosome storage disorder (LSD) genes [9, 10]. Among the various genetic factors identified, the glucocerebrosidase gene (*GBA1*) is emerging as the predominant hereditary contributor to PD. *GBA1* encodes the lysosomal enzyme glucocerebrosidase (GCase), which is responsible for breaking down glucosylceramide (GlcCer) [11]. In this review, we will summarize the genotypic and phenotypic correlations, etiological mechanisms, biomarkers, and therapeutic approaches for *GBA1*-PD as well as current challenges in its studies. Advances in *GBA1*-PD neuroimaging will not be included here, as they have been extensively reviewed elsewhere [12].

## *GBA1* and Gaucher’s disease (GD)

**Fig. 1:**
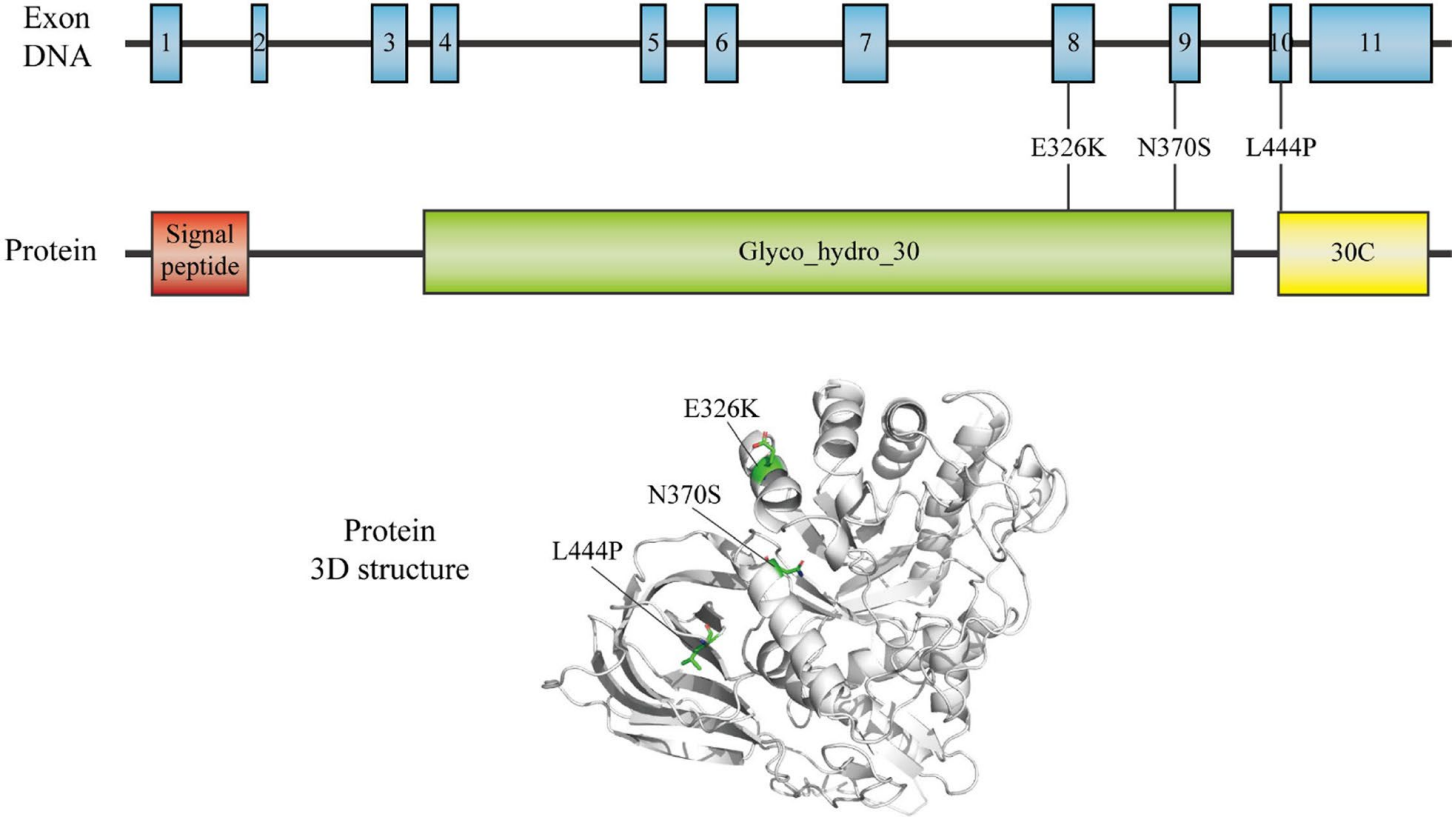
Schematic of the *GBA1* gene, the encoded glucocerebrosidase (GCase) protein, and 3D structure of the protein. The most frequent *GBA1* mutations, L444P, N370S, and E326K, are displayed. The 3D structure of the protein was generated using the PyMOL molecular graphics system

**Fig. 2:**
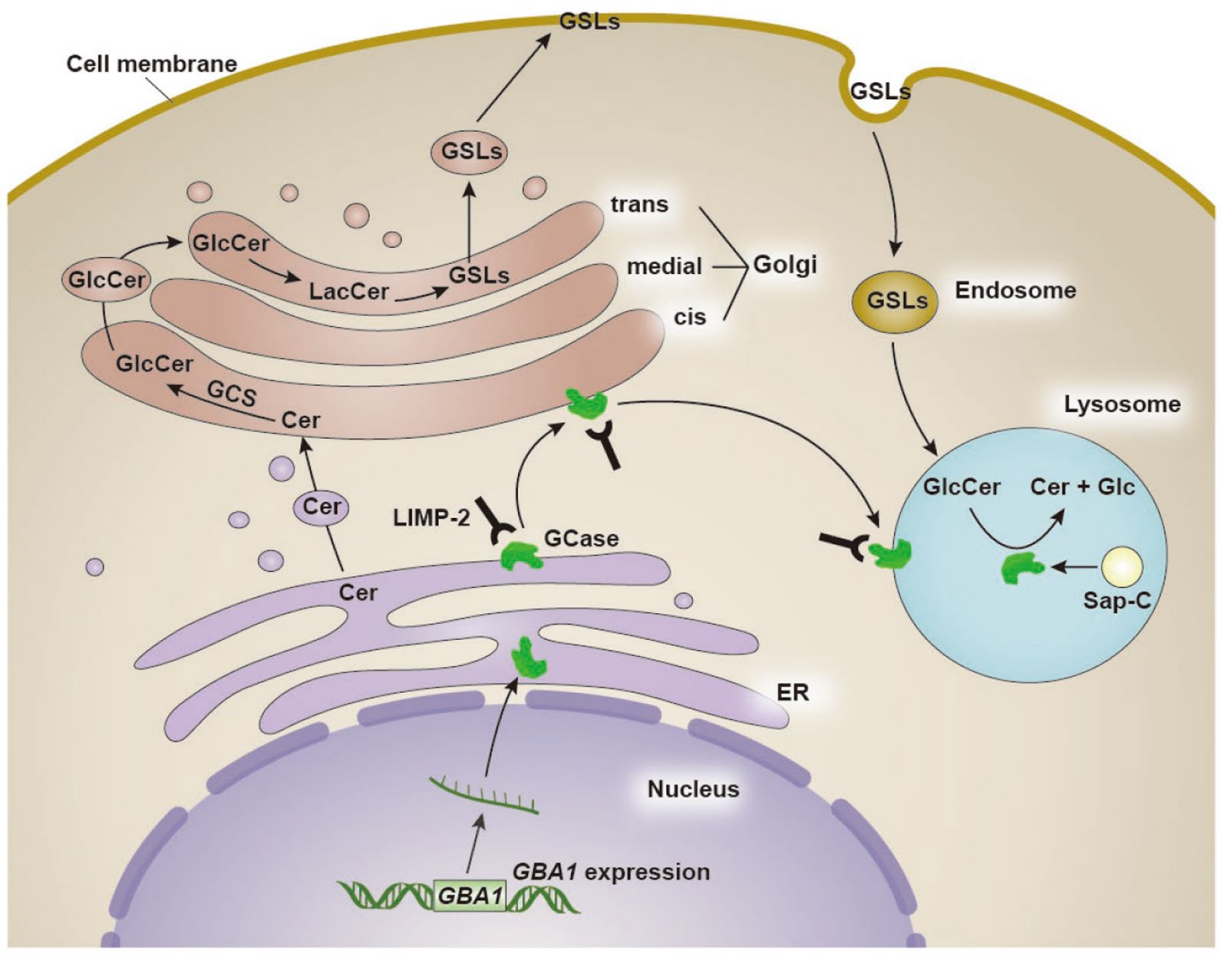
Glucosylceramide (GlcCer) synthesis and degradation pathway. Ceramide (Cer) is synthesized in the endoplasmic reticulum (ER) and then transferred to the cis-Golgi through vesicular transport. Cer is catalyzed by GlcCer synthase (GCS) to produce GlcCer, which is subsequently transported to the trans-Golgi side and transformed to lactosylceramide (LacCer), before being synthesized into complex glycosphingolipids (GSLs). GSLs are delivered to the cell membrane via vesicular transport. GlcCer breakdown is triggered by cell membrane endocytosis. The endosome transports GSLs to the lysosome during endocytosis. Saposin C (Sap-C) activates lysosome glucocerebrosidase (GCase) to degrade GlcCer to Cer and Glucose (Glc)

GD, initially reported by Philip Gaucher in 1882, is a prevalent LSD. The global prevalence of this autosomal recessive genetic disorder is 1.75/100,000. It is rare in the general population and has an impact on 1/40000–1/50000 births; however, it is relatively common among individuals of Ashkenazi Jewish descent, with approximately 1 in every 800 births being afflicted [13]. The pathogenesis of GD is attributed to biallelic variants of *GBA1*. Situated on chromosome 1q21, the *GBA1* gene consists of 11 exons and 10 introns, spanning a length of 7.6 kb, and encodes the protein GCase of approximately 62 kDa, with three structural domains (Fig. 1). The functions and lysosomal targeting of GCase are supported by endogenous transporters and co-factors [14]. Lysosomal integral membrane protein 2 (LIMP2) is a type III transmembrane protein encoded by the *SCARB2* gene. LIMP2 belongs to the CD36 family, is mainly distributed in the limiting membrane of lysosomes and is a receptor involved in the lysosomal translocation of GCase. LIMP2 binds GCase in the endoplasmic reticulum (ER) and reaches the lysosome via the Golgi system, where GCase dissociates from LIMP2 upon reaching the lysosome due to the acidic pH [15–19], where it subsequently interacts with the co-factor saposin C and negatively charged lipids for optimal catalysis activity (Fig. 2) [20]. Mutations in the *GBA1* gene can lead to structural modifications of the GCase protein, resulting in decreased activity, stability, and protein levels of GCase, causing intracellular accumulation of its main substrate, GlcCer, in the reticuloendothelial system, which in turn gives rise to a spectrum of clinical symptoms, such as hepatosplenomegaly, anemia, thrombocytopenia, bone abnormalities, and neurodegeneration [21].

Globally, approximately 70%–98% of GD is caused by five relatively common mutations in *GBA1*, N370S, L444P, 84GG, IVS2 + 1 and RecNcil [22]. In GD, GCase activity is typically 10%–20% of that in normal individuals [23]. Different mechanisms can lead to reduced GCase activity, such as a loss of transcription/translation, misfolded GCase proteins that promote ER stress and activation of the unfolded protein response (UPR), inability of GCase to leave the Golgi apparatus, or loss of key amino acids in its catalytic domains. Similarly, different *GBA1* variants may affect GCase activity through different mechanisms. For example, the L444P and N370S variants are associated with UPR activation and ER stress and may disrupt binding between GCase and saposin C, preventing saposin C activation of GCase [24–27]. The 84GG insertion is a frameshift mutation that typically results in premature stop codons and severely truncated or abnormally long proteins, leading to very low or even completely eliminated GCase activity [23].

GD exhibits a wide range of phenotypes that can be viewed as a spectrum, ranging from asymptomatic forms to severe organ damage and severe neurologic complications. Depending on the neurologic impairment, GD can be categorized into three subgroups. Type 1 is the predominant, non-neuronopathic form that may occur at any age. Types 2 and 3 generally have a more pronounced severity, often accompanied by different levels of neurologic damage [13]. These two subtypes are often referred to as neuronopathic GD. The acute neuronopathic type 2 GD is the rarest and most severe form. Patients with this type of GD usually die before the age of 2 years. The chronic neuronopathic type 3 GD is an intermediate form as it impairs functions of the central nervous system more slowly and gradually than type 2. Nonetheless, the entire spectrum of GD encompasses a diverse array of clinical manifestations, and neurologic impairment can manifest throughout the course of the disease [28].

## *GBAP1*

Pseudogenes are DNA sequences with a high degree of homology to functional genes. Even though pseudogenes are considered ancient genes that have lost their function, they are still involved in the regulation of the expression of their parental genes at the transcriptional and translational levels [29]. The pseudogene of *GBA1*, *GBAP1*, is located approximately 16 kb downstream of this gene with a length of 5.7 kb [29, 30]. The coding region of the *GBA1* gene shares 96% homology with the exonic region of *GBAP1*, while the region between the intron 8 and 3′ untranslated region has as high as 98% homology. The main exonic difference is a 55-bp deletion in exon 9, and the sequence similarity makes *GBA1-*specific sequencing difficult [31, 32]. The high degree of homology and proximity between *GBA1* and *GBAP1* facilitates recombination events caused by gene conversion, fusion, or duplication, and can give rise to a variety of structural variants [33, 34]. The recombination events between *GBA1* and *GBAP1* result in several different Gaucher mutations and mutant groups. The highest homology between *GBA1* and *GBAP1* is between exons 8 and 11; thus, the majority of disease-causing mutations are clustered at this position [35, 36]. Pseudogenes can act as competitive endogenous RNAs (ceRNAs) for their parental genes and compete for regulatory microRNAs (miRNAs). *GBAP1* may act as a ceRNA to regulate *GBA1* expression through miRNAs [37, 38]. The complexity of *GBA1* gene rearrangements cannot be adequately captured using most current next-generation sequencing (NGS) methods, and Sanger sequencing should be used to achieve most accurate genotyping. Long-read NGS has improved ability to distinguish functional genes such as *GBA1* from their pseudogenes; however, currently, its ability to identify recombinant alleles is still limited [39]. It is believed that with the rapid development of sequencing technology, the detection of *GBA1* variants will be improved continuously [39, 40].

## *GBA1* associated with PD

Since the 1980s, clinicians have observed that many individuals diagnosed with type 1 GD exhibit concurrent PD, prompting investigations into the potential role of the *GBA1* gene as a hereditary contributor to PD [41]. Over time, researchers have found that both biallelic and monoallelic mutations in *GBA1* can increase the susceptibility to PD [42–44]. In 2009, a comprehensive meta-analysis involving 16 centers worldwide revealed an odds ratio of 5.43 for any *GBA1* mutation between PD patients and the control group, underscoring *GBA1* variants as a common risk factor for PD [45]. *GBA1* mutation carriers face a significantly heightened rate of developing PD, ranging from 5 to 30 times [45–48], and this risk is influenced by factors such as age, ethnicity, and mutation type, with carriers of severe *GBA1* mutations having a 3- to 4-fold higher risk than carriers of mild *GBA1* mutations [48, 49]. Notably, there are disparities in the distribution frequency of *GBA1* alleles among different ethnicities, with *GBA1* variants accounting for 20%–30% of Ashkenazi Jews and 5%–15% of European patients [50]. In Chinese populations, *GBA1* variants are found in 5.4%–8.4% of individuals with PD, compared to less than 1% in the general population [51–54].

## Classification of *GBA1* variants

Currently, over 300 *GBA1* mutations and gene rearrangements have been reported. The classification of *GBA1* variants is mainly based on the GD phenotype caused by the mutation. Mild mutations are typically associated with type 1 GD, whereas severe mutations lead to types 2 and 3 GD. Certain variants of the *GBA1* gene do not result in GD when present in homozygous carriers; however, these variants elevate the susceptibility to PD and are therefore categorized as “risk” variants. Additionally, a growing number of “unknown” variants have been reported, owing to the limited knowledge of their pathogenicity concerning GD or PD. Overall, 5.9% are classified as mild (e.g., N370S, R496H), 22.6% as severe (e.g., L444P, 84GG, IVS2 + 1, V394L, and D409H), and 0.8% as risk (E326K, T369M, and E388K), with the remaining categorized as unknown (e.g., A456P, K(-27)R, R39C, and R44C) [49]. It is important to note that the relationships between genotype and phenotype are not universally applicable, given that there are some outliers. Parlar et al*.*[49] recently compiled an inventory of all *GBA1* mutations found in patients with PD and developed an online viewer (https://pdgenetics.shinyapps.io/gba1browser/) to search for these variants. The browser summarizes all reported *GBA1* variants in PD to date, and in addition to classifying the variants as described above, they also categorize the pathogenicity of *GBA1* variants according to the American College of Medical Genetics and Genomics (ACMG) classification of variant in GD (Additional file 1: Table S1). Notably, the distribution of *GBA1* variants varies significantly in different ethnic groups. The most common *GBA1* mutation in PD among Ashkenazi Jewish is the N370S mutation. East Asians and South Asians predominantly exhibit the L444P mutation, while Europeans have a higher prevalence of the E326K and T369M *GBA1* mutations [51, 55–57].

## Penetrance of *GBA1* mutations

Penetrance is the ratio of all individuals carrying a disease-associated allele to those affected by the disease. Large-scale sequencing and genotyping studies provide a powerful means to understand the penetrance of pathological mutations/genotypes. The main methods used to estimate penetrance of mutations in PD are the Kaplan–Meier method for unrelated individuals and the kin-cohort method within families [58]. The penetrance of *GBA1* increases with age, with the reported penetrance for *GBA1* heterozygous mutations ranging from 10% to 30% at 80 years of age, suggesting that the majority of *GBA1* heterozygous mutation carriers will never develop PD [59–61]. Similarly, follow-up of nine *GBA1* L444P/R non-manifesting carriers for up to 10 years did not find any of them developing PD [62]. Notably, a study in patients with Ashkenazi Jewish heritage showed an increased risk for PD in both patients with GD (carrying 2 mutant *GBA1* alleles) and heterozygote carriers (carrying 1 mutant *GBA1* allele); however, the overall risk for developing the disease was similar between the two groups of patients, suggesting that the number of mutant alleles may not affect the penetrance [43].

The reason for the relatively low rate of PD phenotypic transformation in individuals carrying *GBA1* mutations remains elusive. Genetic factors may be involved. The *GBA1*-associated risk is significantly influenced by the genetic risk score. Specific locus variants, such as *SNCA* and *CTSB*, could also influence the risk of PD by interacting with *GBA1*. In addition, environmental, aging, and epigenetic factors are also involved [38, 63, 64]. Therefore, in studies targeting *GBA1* penetrance, a comprehensive assessment of genetic factors should be performed [63, 65], taking into account the influence of non-genetic factors such as environmental factors, aging, demographics/ethnicity and diet. Large prospective cohort studies and meta-analyses in *GBA1* non-manifesting carriers would allow for more accurate estimates of *GBA1* penetrance [43].

## Genotype–phenotype characteristics of *GBA1*-PD

**Fig. 3:**
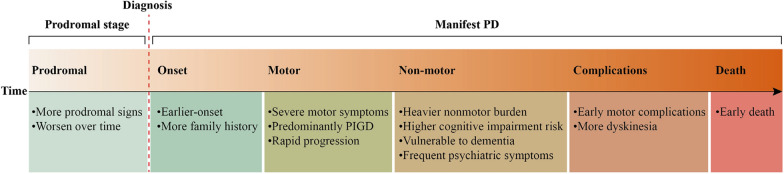
Stage characteristics of *GBA1*-PD. PIGD, postural instability and gait difficulties

The relationship between genotype and phenotype is complex. Although the general phenotype of *GBA1*-PD resembles idiopathic PD, patients with *GBA1*-PD exhibit distinct clinical features as a group [66]. *GBA1* mutations accelerate the neurodegenerative process, resulting in significantly impaired dopaminergic functions and a more severe clinical phenotype [67, 68]. Specifically, *GBA1*-PD tends to have an earlier onset [55, 69], is more likely to have a family history, and is associated with more severe motor and non-motor symptoms. In addition, patients with *GBA1*-PD have more rapid disease progression and higher mortality (Fig. 3) [70, 71].

A dose-dependent relationship between *GBA1* variants and the age of PD onset may exist. Compared to heterozygous and mild or risk variant carriers, carriers of biallelic *GBA1* mutations (either homozygous or compound heterozygous) or severe variants tend to have an earlier age of onset [43, 48, 72, 73]. Prodromal symptoms of PD may be more prevalent among *GBA1* variant carriers [28, 74]. *GBA1* non-manifesting carriers may present with typical prodromal symptoms of PD, such as rapid eye movement sleep behavior disorder (RBD), hyposmia, cognitive decline, and fine motor symptoms, and these symptoms may progressively worsen over time [28, 75–80].

Individuals harboring *GBA1* mutations exhibit an increased incidence of motor complaints and a higher likelihood of postural instability and gait difficulties, along with gait and balance disorders [81]. They often experience more rapid progression of motor symptoms [82]. Additionally, patients with *GBA1*-PD experience motor fluctuations and dyskinesias earlier [47, 73, 83]. In terms of non-motor symptoms, patients with *GBA1*-PD are more susceptible to developing RBD and autonomic complaints such as constipation, and a greater degree of olfactory loss [55, 68, 73, 79, 84, 85]. They are six times more likely to develop dementia than non-carriers, and have more rapid progression of cognitive impairment [68, 71, 73, 86–88], particularly in visuospatial ability, executive function, and working memory [89, 90]. The more extensive neocortical Lewy body-type lesions in patients with *GBA1*-PD, and the higher rate of *GBA1* mutation in patients with dementia with Lewy bodies (DLB), also reflect, to some extent, the close relationship between PD and dementia [91, 92]. Additionally, there is an increased prevalence of psychological symptoms, including hallucinations, delusions, and impulsive behavior in patients with *GBA1*-PD [84]. In terms of outcomes, *GBA1* mutation carriers have twice the risk of death than non-carriers, die 1 year earlier, and may have lower survival rate when carrying severe mutations [71].

## Modification of *GBA1*-PD by other genes

Patients carrying both *LRRK2* and *GBA1* gene variants have a milder phenotype and exhibit fewer motor, non-motor, and dementia symptoms compared to patients carrying a single variant allele of *GBA1*, suggesting that *LRRK2* gene mutations may have a beneficial effect in individuals with *GBA1* gene mutations [93, 94]. However, the exact underlying mechanism remains incompletely understood. One possible explanation is the presence of crosstalk between the two proteins. In dopaminergic neurons derived from PD patients with *LRRK2* mutations, GCase activity is diminished, and inhibition of the kinase activity of LRRK2 leads to increased GCase activity, with the LRRK2 substrate Rab10 as a key mediator in the regulation of this process [95]. Furthermore, inhibition of LRRK2 kinase reverses some lysosomal impairments and inflammatory responses observed in astrocytes with *GBA1* mutations [96].

Genes closely associated with Alzheimer’s disease (AD) can also influence the phenotype of *GBA1* carriers. For example, individuals carrying mutations in both *GBA1* and the *APOE* epsilon 4 allele show a higher risk for cognitive impairment [90]. This combination results in a five-fold elevation in the likelihood of developing PD dementia and accelerates the rate of cognitive deterioration [97]. Bridging Integrator 1 (*BIN1*), a second major risk locus for late-onset AD, plays a crucial role in mediating tau pathology, endocytosis, inflammation, calcium homeostasis, and apoptosis [98–100]. Alterations in the *BIN1* locus may also influence the age of onset of *GBA1*-PD [101].

It is important to recognize that the genotype–phenotype relationships are complex and highly heterogeneous. To fully understand the impact of *GBA1* variants on the natural history of PD, it is necessary to examine the prodromal symptoms of PD in *GBA1* non-manifesting carriers. Long-term prospective cohort studies of such risk groups can help identify clues to the transformation of PD. Evaluating the impact of *GBA1* variants on various types of motor and non-motor symptoms, motor complications, death, and other outcomes of PD is significant to elucidate the trajectory of *GBA1*-PD. Considering the impact of different mutation types on the *GBA1* phenotype, it is necessary to stratify patients according to the *GBA1* mutation type, include more homozygous and complex congenic carriers (GD-PD), and exclude common PD-associated genes such as *LRRK2* and *PRKN* from clinical trials [71, 73, 82, 102, 103]. Additionally, it is necessary to consider the impact of medication factors and the on/off status of clinical symptoms when evaluating patients. In addition to clinical scale assessments, the use of highly specific instruments or objective measures with cutting-edge technologies such as biochemical, neuroimaging, and artificial intelligence approaches will improve the sensitivity to subtle changes [61, 104, 105]. It is worth noting that the disease phenotype is influenced by a variety of factors, such as the environment, different protein expression thresholds, or other modifier genes. Only by comprehensively considering these important phenotypic regulators can the complex relationship between genotype and phenotype be finally unraveled [25, 106].

## Mechanism of *GBA1* involvement in PD

**Fig. 4:**
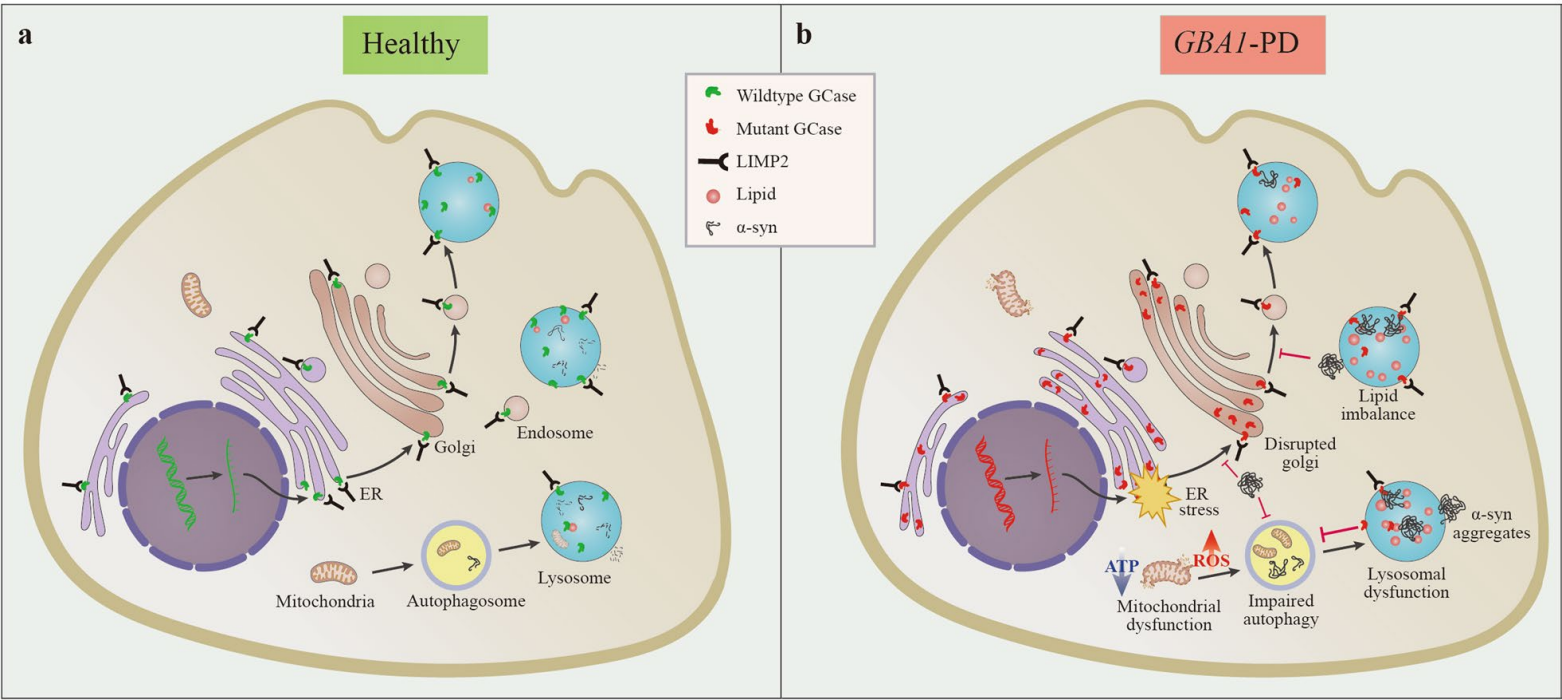
Mechanism of mutant glucocerebrosidase (GCase) in PD. **a** Under healthy conditions, mRNA from the *GBA1* gene travels from the nucleus to the endoplasmic reticulum (ER). GCase is produced in the ER, transferred to the Golgi apparatus by binding to the protein LIMP2, and then fused with lysosomes, where GCase is activated to exert its hydrolytic effect. Usually, α-syn can be phagocytosed by autophagosomes and then targeted to lysosomes, where GCase interacts with α-syn to promote its degradation. **b** *GBA1* mutations lead to impaired GCase activity, and GCase proteins misfold and lodge in the ER, initiating ER stress. Lack of GCase in lysosomes impairs the autophagic lysosomal pathway (ALP), causing accumulation of lipid substrates such as GlcCer in lysosomes and α-syn aggregation. This accumulation prevents GCase transfer from the ER/Golgi to the lysosome, exacerbating lysosomal dysfunction. GCase deficiency and reduced ALP function may cause mitochondrial dysfunction, resulting in reactive oxygen species (ROS), decreased ATP generation, and aberrant mitochondrial morphology. In addition, GCase deficiency, accumulation of lipids, and α-syn may activate microglia and cause neuroinflammation. All of these contribute to cell death and the development of PD

In recent years, research has delved into the role of mutant *GBA1* in the pathogenesis of PD. *GBA1*-PD does not strictly adhere to Mendelian laws, as both gain-of-function and loss-of-function mutations have been associated with an elevated risk of developing PD. The mechanisms underlying the increased risk of PD from *GBA1* variants remain incompletely understood. Research progress has indicated the involvement of dopaminergic neuronal damage, α-syn aggregation, lipid metabolism disorders, autophagy-lysosome dysfunction, ER stress, mitochondrial damage, and inflammatory responses (Fig. 4). In the following, we will discuss each of the listed mechanisms separately in detail.

### GCase and dopaminergic neurons

A central question regarding *GBA1*-related PD is how the loss of GCase activity predisposes dopaminergic neurons to the accumulation of α-syn [107]. Although there may be a bidirectional feedback loop between GCase deficiency and α-syn accumulation, it does not explain the selective vulnerability of midbrain dopaminergic neurons [108]. However, there is much evidence to support a strong link between *GBA1* variants and dopaminergic neurons. For example, the GCase enzyme activity was found to be reduced in dopaminergic neurons from patients with *GBA1*-PD, and there is a significant loss of dopaminergic neurons in the brain [107, 109, 110]. In *Drosophila*, mutant GCase and α-syn have synergistic deleterious effects on dopaminergic cells [111]. Studies using many cellular/neuronal models have also shown that *GBA1* mutations can exacerbate the bioenergetic burden on dopaminergic neurons via disruption of lipid homeostasis, release of inflammatory factors, oxidative stress, mitochondrial dysfunction, and impaired calcium homeostasis [107, 108, 112, 113]. However, downregulation of GCase expression alone is not sufficient to cause the key pathological features of PD in vivo. Synergization with α-syn overexpression is required for GCase deficiency to drive nigrostriatal neurodegeneration [110]. A study by Henderson et al. [114] showed that reduced GCase activity alone does not lead to the aggregation of α-syn, but rather functions by enhancing the aggregation of pre-existing misfolded α-syn, and this modulatory effect is not dependent on the neuronal type. Indeed, α-syn and GCase are widely expressed in the brain, affecting different cell types [108, 115]. Notably, Burbulla et al. [116] showed that although increased α-syn affects GCase transport and lysosomal function in both dopaminergic and non-dopaminergic cells, oxidized dopamine accumulation was only observed in dopaminergic cells, which could reduce wild-type or mutant GCase activity [116–118]. In *GBA1*-PD organoid studies, NURR1, a transcription factor essential for the differentiation, maturation, and maintenance of midbrain dopaminergic neurons, was found to be significantly reduced, and this reduction led to reduced dopamine production and increased susceptibility of these neurons [119]. It has also been hypothesized that mutated GCase proteins do not fold sufficiently in the ER, leading to protein accumulation in the cellular compartment, which induces dopaminergic neuronal response to stress, causing their damage and death [120].

### The vicious cycle between GCase and α-syn

PD-related mutations in *GBA1* may impair GCase activity. GCase deficiency increases the aggregation and spread of α-syn and promotes neurodegeneration in PD. Numerous basic studies have demonstrated the close relationship between GCase and α-syn (Table 1) [114, 121, 122]. Examination of mutations across several vertebrate species indicates a potential co-evolutionary relationship between GCase and α-syn, as alterations in either protein can hinder their interaction. Decreased GCase activity may lead to oligomerization of α-syn aggregates, and accumulated α-syn may also impede GCase migration from the ER to the lysosome, resulting in a decrease in GCase activity, leading to a pathogenic feedback loop [114, 121, 123].

**Table 1:** Cellular and animal studies of the relationship between GCase and α-syn

Study type	Effect of GCase on α-syn	Effect of α-syn on GCase
Cellular studies	Elevated α-syn was observed in *GBA1*-PD-derived iPSC neurons [107] and midbrain organoids lacking GCase [272]	Reduced GCase activity in differentiated dopaminergic SH-SY5Y cells or hippocampal and cortical neurons treated with preformed α-syn fibrils [114, 273]
	Elevation of α-syn in cells after GCase inhibition with CBE [274]	Oligomeric α-syn inhibited GCase activity more efficiently than monomeric α-syn [275]
	Reduced GCase activity and elevated total α-syn levels in *GBA1*-knockdown cells were restored by treatment with recombinant GCase [276, 277]	Overexpression of α-syn reduced GCase activity in SH-SY5Y cells [187]
	Exogenous wild-type GCase reversed the elevation of intracellular α-syn caused by decreased GCase [278]	
Animal studies	In *Gba1* L444P heterozygous mice, GCase dysfunction enhanced neuronal vulnerability and dopaminergic cell loss triggered by increased α-syn [218]	Increased GCase activity in α-syn knockout mice [279]
	Inhibition of GCase with CBE resulted in increased oligomeric α-syn levels in selected brain regions [280]	
	GCase inhibited α-syn aggregation in the brains of A53T α-syn mice [279, 281]	
	Brain tissues of *Gba1* knockout mice showed aggregation of α-syn [121, 282]	

α-syn, alpha-synuclein; CBE, conduritol B epoxide; GCase, glucocerebrosidase; *GBA1-*PD, Parkinson’s disease with *GBA1* mutations; iPSC, induced pluripotent stem cell

Additionally, α-syn can directly interact with GCase under acidic conditions, further inhibiting GCase function [124]. Reduced GCase activity can potentially enhance α-syn phosphorylation at serine 129 and oligomerization in the brains of aged monkeys [125]. Various risk variants of *GBA1* may result in reduced GCase activity through several mechanisms, including direct reduction of enzyme activity, failure to adhere to the ER quality control systems resulting in proteasome degradation, retention within the ER or Golgi apparatus, and incorrect ligation of LIMP2 or saposin C [25, 126]. Studies conducted on human samples have revealed a negative correlation between pathological α-syn and GCase activity in brain tissues of PD patients and a negative correlation between GCase activity in leucocytes and plasma oligomeric α-syn in patients with GD [123, 127, 128]. DLB, characterized by the accumulation of aggregated α-syn protein in LBs and Lewy neurites as the main pathologic features, is also strongly associated with GCase, which has been detected in LBs [129, 130]. Decreased GCase activity and elevated glycosphingolipid (GSL) level may exacerbate Lewy pathology [131]. These findings collectively support the close relationship between α-syn and GCase. However, it is important to note that the incidence of PD does not appear to increase with decreased enzyme activity, and a considerable proportion of patients with GD never progress to PD. Therefore, considering that the loss of enzyme activity is not the only cause of PD, further investigation is needed to explore other mechanisms by which *GBA1* mutations contribute to α-syn accumulation.

### Lipid metabolism disorder

GSLs are found in cell membranes and play a critical role in maintaining membrane function and facilitating cellular communication in a variety of tissues, including the brain. However, they are not restricted to the intracellular compartment, but also act in the extracellular space (e.g., plasma) through unknown mechanisms. GlcCer and glucosylsphingosine (GlcSph), substrates of GCase, both belong to the class of GSLs. GlcCer is abundant in the brain and plays an essential role in intracellular membrane trafficking, signal transduction, and cell proliferation [132, 133]. Mutations in the *GBA1* gene affect the metabolism of GSLs, and dysfunction of the enzyme GCase results in the intracellular accumulation of its upstream substrate GlcCer, which can leave the lysosome to enter the cytoplasm or remain in the lysosome and be converted to GlcSph and released into the cytoplasm [134, 135]. Sphingolipids, including GlcCer and GlcSph, have been reported to affect the secondary structure and aggregation kinetics of α-syn under acidic or neutral pH conditions in vitro and to exacerbate Lewy pathology by affecting the fluidity of lysosomal membranes and promoting the formation and aggregation of α-syn fibers [121, 131, 136–143]. Conversely, oligomers of α-syn impede GCase migration from the ER to the Golgi, reduce GCase activity, and affect substrate accessibility and turnover, leading to further accumulation of GlcCer [121, 144, 145].

Animal studies have shown that reducing GSLs with GlcCer synthase inhibitor slows the progression of synucleinopathy [146]. Despite increasing data on the effects of *GBA1* on lipid metabolism and PD pathogenesis, there is still a long way to go to fully understand the complex interactions between genetic background, disease status, GCase activity, lipid metabolism, and protein pathology.

### Impaired autophagy-lysosome pathway (ALP)

Lysosomes are acidic organelles that degrade toxic cellular contents through enzymatic degradation and autophagic pathways. Many neurodegenerative disorders, including PD, are associated with lysosomal abnormalities. Autophagy is the cellular process through which an organism removes proteins and degrades dysfunctional organelles within the cell, which may prevent cytotoxicity and cell death. Since α-syn undergoes degradation within lysosomes, dysfunction in the ALP could hinder the clearance of α-syn, leading to its aggregation into harmful oligomers. Consequently, this can exacerbate the cycle of impaired autophagy and lysosomal function [11, 147]. Evidence from experimental PD models suggests a strong association between *GBA1* and ALP regulation [95, 148].

The autophagy pathway can be categorized into macroautophagy, microautophagy, and chaperone-mediated autophagy (CMA). Macroautophagy is the primary regulated type of autophagy that responds to environmental and physiological cues, microautophagy involves the direct engulfment of cytoplasmic contents by lysosomes through membranous invaginations, and CMA is a protein-exclusive type of autophagy involving chaperone-assisted translocation of substrate proteins across the lysosomal membrane [149, 150]. Macroautophagy and CMA are particularly relevant to PD [151]. Cells and animal models deficient in GCase show impairment of ALP, with decreased lysosomal GCase activity for macroautophagy and CMA and interfering with α-syn clearance [152]. Mice deficient in the lysosomal enzyme β-hexosaminidase exhibit α-syn accumulation in neurons [153]. Studies of fibroblasts from PD patients carrying the N370S *GBA1* variant revealed increased autophagic structures in the variant cells and accumulation of SQSTM1/p62 [154]. PD patients exhibit CMA defects in their brains, with autopsies indicating a significant decrease of CMA markers in specific regions and accumulation of autophagy-associated proteins in the LBs [155]. Single-cell transcriptional analyses and proteomics have confirmed decreased CMA activity and proteomic alterations in *GBA1*-PD [156]. Modulating autophagy can ameliorate disease pathology. For example, inducing autophagy using the autophagy activator rapamycin or promoting GCase translocation into lysosomes with the GCase chaperone isofagomine can reverse α-syn accumulation [157]. Overexpression of Beclin 1, which stimulates autophagy, can ameliorate the neurodegenerative pathology in PD animal models [158].

In addition to macroautophagy and CMA damage, GCase depletion has been found to cause lysosomal dysfunction in mouse neurons and *Drosophila* brains [121, 159]. *GBA1* D409V-knock-in mouse astrocytes exhibit extensive defects in lysosomal morphology and function, including reduced lysosome numbers, fewer acidic vesicles, and reduced lysosomal degradative activity [146, 148]. GCase-deficient *Drosophila* exhibit many abnormally expanded lysosomes in the brain [160]. The mechanisms by which the loss of GCase activity affects lysosomes are unclear. GCase dysfunction impairs the lysosomal compartment, establishing a lysosome–plasma membrane axis that leads to structural changes of the plasma membrane and alterations of intracellular signaling pathways. At the same time, accumulation of GlcCer or GlcSph, substrates of GCase, could contribute to lysosomal dysfunction and toxicity [161, 162].

Studies in PD patients have revealed extensive lysosomal dysfunction. Variants in LSD genes have been detected in both PD and *GBA1*-PD patients [163], and LSD patients (GM1 and GM2 gangliosidosis, neuronal ceroid lipofuscinosis, and Fabry disease) can exhibit typical PD symptoms [164]. To date, many genetic loci related to autosomal/lysosomal function have been identified as risk factors for familial and sporadic PD. Large-scale meta-analyses of GWASs have implicated several dozens of loci associated with lysosomal functions and pathways in PD pathogenesis [5, 6, 165–167]. Multiple lines of evidence suggest that genetic variants of lysosomal genes contribute to PD more broadly. In addition to *GBA1*, other genes involved in LSD (e.g., *SMPD1*, *SCARB2*, and *GALC*) can also increase the risk of PD. Other lysosomal genes (*ATP13A2*, *LAMP1*, *TMEM175*, and *VPS13C*) that increase the burden of variants in PD patients have recently been reported [163, 168–171]. Growing evidence also highlights the impact of multifunctional lysosomal proteins (e.g., LIMP2, prosaposin, progranulin, and cathepsin D) on PD [172]. LIMP2 deficiency reduces GCase activity, impairs autophagy/lysosomal function, and leads to lipid storage and α-syn accumulation, resulting in neurodegeneration, inflammation, and apoptosis. Conversely, overexpression of LIMP2 reduces α-syn levels [173]. Changes in lysosomal enzymes associated with various degradation pathways have been observed in body fluids and tissues, including the brain, the cerebrospinal fluid, and fibroblasts of PD patients [131, 174–178]. For example, expression of the major lysosomal protease cathepsin D is reduced in nigral neurons in PD patients, especially in neurons containing α-syn inclusions [179]. At the same time, altered activities of cathepsin D, cathepsin E, α-fucosidase, β-hexosaminidase, and β-galactosidase are observed in the cerebrospinal fluid of patients with PD [175–177].

### ER stress

ER stress may cause several neurodegenerative disorders. *GBA1* abnormalities can also induce PD through ER dysfunction. ER is essential for protein folding, lipid synthesis, and calcium storage. Dysregulation of ER homeostasis can activate the UPR, initiating a cascade of significant stressors within the ER. This response aims to restore the equilibrium but may activate pathways that lead to cellular demise [180].

GCase is synthesized on polyribosomes bound to the ER and subsequently translocated into the ER. Within the ER, the protein undergoes N-linked glycosylation at four asparagine residues and quality control [120]. Mutant GCase proteins are misfolded, resulting in varying degrees of ER retention, triggering ER stress, UPR, and Golgi fragmentation [181]. UPR and ER stress can result in autophagy damage, increased apoptosis, and lysosomal insufficiency, weakening the protective role of autophagy against synucleopathies and ultimately contributing to PD development [154]. ER stress and UPR activation markers are elevated in *GBA1*-mutant brain tissues as well as cellular and animal models [182–186], supporting the conclusion that *GBA1* mutations cause abnormal changes in ER function. In α-syn-overexpressing neurons, the inability of LIMP2 to bind to GCase leads to increased GCase retention in the ER and decreased GCase activity in the lysosome [121, 187].

### Mitochondrial dysfunction

Mitochondrial turnover is essential for neuronal protection. Failure to remove damaged mitochondria can lead to the accumulation of reactive oxygen species (ROS) and free radicals, causing neural damage. The onset of PD is associated with mitochondrial dysfunction, and PD-associated genes are linked to mitochondrial morphology and function. Increasing evidence shows that decreased GCase activity may disrupt mitochondrial function [188–190].

Impaired mitochondrial function and morphology, reduced ATP production, and increased oxidative stress have been observed in GCase-deficient cells, animal models, and PD patients [189, 191, 192]. In SH-SY5Y neuroblastoma cells, inhibition of GCase activity by conduritol B epoxide (CBE) significantly reduced mitochondrial membrane potential (Ψm). Ψm, generated by the mitochondrial electron transport chain, plays a significant role in ADP phosphorylation into ATP [193]. GD fibroblasts show mitochondrial abnormalities associated with impaired macroautophagy flow [194]. Mitophagy activation decreases the colocalization of mitochondria and LC3 in *GBA1*-knockout cells [195]. Abnormal mitochondrial morphology and function, increased ROS, reduced ATP production, autophagosome accumulation, and impaired mitophagy have been observed in *GBA1* L444P heterozygous mutant mouse models, *GBA1* knockout mice, and GCase-deficient *Drosophila* brains, findings that are consistent with those observed in patients with *GBA1*-PD [186, 196–198]. Mitochondrial accumulation of α-syn was seen in cortical neurons in GD mice and resulted in abnormal mitochondrial morphology and function [199].

### Inflammatory response

*GBA1* mutations may be associated with inflammation [200–202]. GD is accompanied by significant alterations associated with inflammation, including elevated proinflammatory factors and chemokines, and activation of microglia, astrocytes, and T cells [203]. Reduction of GCase activity by CBE treatment in animals induced strong neuroinflammatory responses, complement activation, microglial activation, and insoluble α-syn aggregation [204–206]. Similarly, inflammatory mediators are increased in the plasma from *GBA1*-PD patients [207], and levels of interleukin (IL)-8, monocyte chemoattractant protein-1, and macrophage inflammatory protein-1 alpha are significantly associated with the *GBA1* mutation genotype [208]. Macrophages from patients harboring the homozygous *GBA1* N370S mutation exhibit increased secretion and inflammatory activation of IL-1β and IL-6 [209]. GCase activity inhibition, α-syn aggregation, and monocyte lineage-mediated inflammation lead to pathogenic cascade responses. These responses can induce neuronal damage and cell death and ultimately drive PD pathology [210].

## Advances in cellular and animal models of *GBA1*-PD

Establishing suitable models to study the pathogenesis of PD remains a challenging task. Human induced pluripotent stem cell (iPSC) technology provides an opportunity to study the role of genetic mutations in the pathogenesis of PD. The iPSC technology enables studies of patient-derived cells in the genetic context of the patient’s disease [108]. Isogenic gene-corrected cell lines help uncover the genetic mutation-associated abnormalities that are involved in disease onset and neuronopathic phenotypes [211, 212]. Cell models generated from neural derivatives of iPSCs based on *GBA1* mutations provide a unique tool for studying pathological changes within PD cells [213]. Another in vitro approach for studying human brain functions is the use of brain organoids. Compared to standard 2D cultures, iPSC-derived 3D organoids have greater similarity to the in vivo cellular tissues and organ structures, allowing for the assessment of specific mutations in more complex neural-specific models [25, 119, 214]. Patient-derived midbrain organoids carrying specific genetic mutations represent a more suitable tool to generalize the disease process occurring in PD patients [215, 216]. Midbrain organoids mimic early embryonic neurodevelopment and are ideal models for studying PD-related neurodevelopmental phenotypes. Midbrain organoids with *GBA1* N370S mutation show severely impaired neurodevelopment [119]. In terms of animal models, *GBA1*-associated PD models have been developed in combination with overexpression of α-syn [217–219]. Progranulin (PGRN), encoded by *GRN*, is a novel GCase modifier. In vivo and in vitro data suggest that PGRN deficiency plays a crucial role in the initiation, progression, and regulation of GD associated with *GBA1* mutations. Some researchers have crossed PGRN-deficient mice (*Grn*^−/−^) with *Gba*^D409V/D409V^ (*Gba*^9v/9v^) mice to produce *Grn*^−/−^*Gba*^9v/9v^ (termed PG9V) mice. This mouse model overcomes many of the limitations of existing models, as it exhibits the neurodegenerative manifestations of GD and PD, with an exacerbated GD phenotype [220].

## Biomarker studies of *GBA1*-PD

Objective biomarkers for *GBA1*-PD are still lacking, but dysfunction in the GCase pathway offers insights into potential biomarkers for PD. GCase activity and sphingolipid content can be determined from specimens such as brain tissue and blood. These biomarkers can potentially improve patient management and counseling in clinical settings, and serve as metrics for evaluating the effectiveness of GCase-targeting therapy [24].

### GCase enzymatic activity

Since 2012, studies using brain autopsy and dried blood spot testing have reported reduced GCase activity in PD patients [131, 176–178, 187, 221, 222]. Since most patients with *GBA1*-PD still have a wild-type *GBA1* allele, GCase activity is only slightly reduced when compared with controls, and the extent of this reduction may be related to the variant classification [221–223]. As seen in brain tissue assays, there is a general decrease in GCase activity in patients with *GBA1*-PD, with the most significant decrease in the substantia nigra [187]. However, findings on GCase activity have been inconsistent and, rather surprisingly, reduced GCase protein level and enzyme activity have also been reported in patients with sporadic PD [123, 131, 174, 175, 187]. Compared to brain biopsy, blood samples can be collected prospectively and longitudinally, and the procedure is less invasive than lumbar puncture. Whole blood samples (GCase is active in leukocytes, not plasma) are important for detecting GCase, with numerous studies utilizing dried blood spots to test GCase activity [222]. In 2015, the first study on dried blood spot GCase activity in *GBA1*-PD was conducted [221], showing that the *GBA1*-PD heterozygotes exhibit lower GCase activity than controls. The enzymatic activity decrease shows a dose effect, with GCase activity decreasing with increased number of *GBA1* variant alleles. However, the exact relationship of enzymatic activity with the severity of *GBA1* mutations and the clinical phenotype, is debated. Similar to the results from brain tissue assays, GCase enzymatic activity in blood is reduced even in PD patients lacking *GBA1* mutations [221, 222]. It is noteworthy that there is cellular heterogeneity in GCase activity, as a significant reduction in GCase activity has been observed in monocytes from PD patients, whereas the GCase activity in lymphocytes is not significantly reduced [224].

#### *LRRK2* affects the GCase enzymatic activity

*LRRK2* mutations are a common genetic etiology of PD. A study examined the GCase activity among carriers of *LRRK2* variants and in *GBA1*-PD patients. Surprisingly, the *LRRK2* G2019S carriers showed higher GCase activity than non-carriers [221]. This phenomenon was also observed in the peripheral blood mononuclear cells (PBMC) of individuals with *LRRK2* mutations [225]. The mechanisms underlying the increased GCase activity in *LRRK2* variant carriers remain unclear. One hypothesis suggests that *LRRK2* mutations might result in the expansion of lysosomal compartments, a phenomenon associated with increased GCase activity [226]. Alternatively, *LRRK2* mutation could impact reverse retromer function, potentially altering GCase localization and turnover [222, 227]. However, a recent study on GCase activity in brain tissues did not show any significant changes in the enzymatic activity in PD patients with *LRRK2* mutations [131]. Conversely, another study found decreased GCase activity in the fibroblasts and dopaminergic neurons derived from iPSCs from *LRRK2* mutation carriers, and this reduction could be reversed by inhibiting *LRRK2* kinase [95]. Thus, the *LRRK2* kinase activity affects GCase activity differently depending on the test medium, the cell type, and the technique employed.

### Sphingolipid metabolism

Sphingolipid metabolism can be examined in plasma. However, findings from plasma assays are ambiguous and inconsistent [228, 229]. A pooled data analysis of five studies explored the variability of various GSLs across different samples (plasma, PBMC, and cerebrospinal fluid). Results revealed that elevated plasma levels of GlcCer can help differentiate among idiopathic PD, *GBA1*-PD, and healthy individuals, with the GlcCer C24:1 isoform being the most effective in differentiating *GBA1*-PD from idiopathic PD and healthy individuals [139]. A study of plasma GlcSph, GlcCer, ceramide, and four other lipids in carriers of the *GBA1* N370S mutation found that GlcSph was the only variable associated with *GBA1* mutation status [230]. Several brain tissue studies have found elevated GSLs in idiopathic PD. However, these alterations have been observed with inconsistent conclusions across specific brain regions and age groups. For instance, it has been reported that GlcCer and GlcSph accumulate in non-*GBA1*-PD [229, 231], whereas no accumulation occurs in putamen [162]. It has also been shown that GlcSph levels are elevated in *GBA1*-PD and idiopathic PD, while GlcCer levels are unchanged [131]. Similarly, an analysis of 251 lipids, including 95 sphingolipids, in brain tissues showed changes in ganglioside levels in non-dopaminergic areas of the *GBA1*-PD brain. In contrast, changes in other lipid classes, including GlcCer, were minimal [232]. Conversely, quantitative analysis of sphingolipids in cerebrospinal fluid samples revealed a considerable rise in GlcCer levels and a reduction in the sphingomyelin fraction, a crucial downstream metabolite [233].

In summary, available evidence does not support the use of reduced GCase activity, or GlcCer or GlcSph accumulation as a reliable biomarker of PD. While *GBA1* variant carriers exhibit a trend toward decreased GCase activity in brain tissues, blood and fibroblasts, as well as decreased mean intracerebral GCase activity, there is a substantial overlap between patients and controls. GCase activity is highly variable in populations. Besides the type of *GBA1* mutation, GCase activity is also related to factors such as the sample source and type, sample storage condition and duration, the number of times the samples are frozen and thawed, the assay method used, and other genetic or environmental factors [170, 222, 234–237]. Although the conclusions have not been entirely consistent, current evidence supports the notion that loss of GCase function may be one of the pathogenic mechanisms of PD and is a potential therapeutic target.

There are still many questions about GCase activity, such as the relationship among GCase activity, the disease state, the genetic status, the associated lipid metabolism, and protein pathology in various regions of the brain [131]; and the correlation among GCase activity in dried blood spots, cerebrospinal fluid, and the brain parenchyma. It is important to note that different cells could have different levels of GCase, and cell-based analysis might provide more accurate information for the detection of GCase activity [78, 224, 238]. Further cell-based and animal model studies will be beneficial for elucidating the GCase biology [118]. Likewise, contradictory findings regarding GlcCer and GlcSph concentrations dampen our enthusiasm on their potential as biomarkers for *GBA1*-PD. Some researchers have proposed that since the GCase reaction is part of a complicated sphingolipid cycling pathway, analyzing the consequences of reduced GCase activity on the overall sphingolipid cycling pathway and sphingolipid flux is essential for drawing meaningful conclusions [138]. Investigating cellular pathways beyond GlcCer or GlcSph is necessary to identify the molecular linkage between *GBA1* mutations and PD [232].

## Treatment

The association between *GBA1* variants and PD presents promising opportunities for targeted PD therapies aimed at the GCase pathway. Patients with PD who carry *GBA1* variants often exhibit a more aggressive disease progression phenotype, necessitating more intensive treatment strategies to counteract poor prognosis. The basic principle of clinical trials targeting *GBA1*-PD is to regulate GSL turnover and correct defects in cellular GCase. Specific approaches include enzyme replacement therapy (ERT), substrate reduction therapy (SRT), pharmacological small-molecule chaperones, and gene therapies [24, 126, 239].

### ERT and SRT

**Fig. 5:**
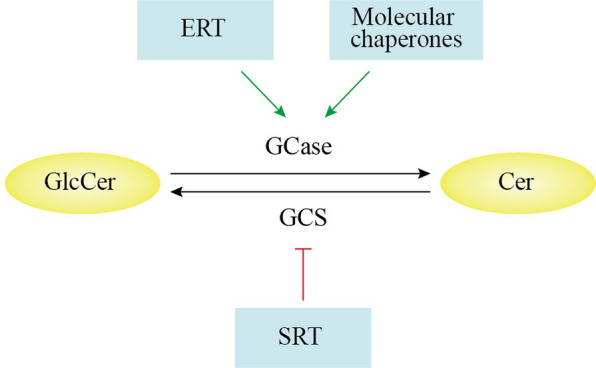
Enzyme replacement therapy (ERT), substrate reduction therapy (SRT), and molecular chaperone therapy targets. Glucosylceramide synthase (GCS) synthesizes glucosylceramide (GlcCer) from ceramide (Cer), and glucocerebrosidase (GCase) degrades GlcCer to Cer. ERT increases GCase, and SRT decreases GlcCer in *GBA1*-PD. Molecular chaperones increase the stability of mutant GCase

ERT and SRT have proven effective therapeutic approaches for GD (Fig. 5). ERT involves administering recombinant GCase enzymes to replace dysfunctional GCase in patients. These enzymes typically possess modified terminal mannose residues, enhancing their targeting and macrophage absorption [180]. However, the recombinant GCase cannot penetrate the blood–brain barrier (BBB), resulting in limited efficacy in addressing neurologic symptoms associated with GD. In contrast, SRT prevents the overaccumulation of GlcCer by inhibiting GlcCer synthase, thereby reducing substrate accumulation [180]. Similar to ERT, SRT also faces challenges in BBB crossing.

Brain-penetrating GlcCer synthase inhibitors such as GZ667161 and venglustat can reduce substrates for GCase enzymes in the brain, decrease α-syn level in experimental animals, and improve animal behavior [240, 241]. Venglustat is safe and well tolerated in PD patients harboring *GBA1* variants (ClinicalTrials.gov ID: NCT02906020, Moves-PD). Venglustat effectively eliminated GlcCer accumulation; however, it did not show a beneficial therapeutic effect [242, 243], suggesting that GlcCer accumulation is not a major pathogenetic mechanism and thus not a desirable pharmacological therapeutic target for PD, and that the complex role of *GBA1* in PD extends beyond substrate accumulation [118, 242, 244, 245]. It is thus necessary to further decipher novel pathway interactions based on GCase–α-syn at the molecular, cellular, and systemic levels.

### Pharmacological small-molecule chaperones

Various small molecules with GCase-activating properties have stabilizing or ameliorating effects on neurological symptoms [246]. More recently, several medications have emerged as potential enhancers of GCase activity [247–251]. Among these, chaperonins are of interest for their ability to enhance lysosomal GCase activity (Fig. 5), as they can penetrate the BBB and correct the folding of GCase in the ER, facilitating its translocation to the lysosome [15]. Preclinical studies have been conducted in *GBA1*-PD models [251–253]. Ambroxol has been shown to enhance GCase activity and is the most promising small-molecule chaperone targeting the GCase pathway [254, 255]. Ambroxol can cross the BBB, and increase GCase level and activity as well as *GBA1* mRNA expression in fibroblasts of patients with PD. It can also increase the expression/activity of lysosomal cathepsins, LIMP2 and saposin C, and modulate multiple cellular pathways to correct lysosomal abnormalities in PD [253, 256–259]. Ambroxol also reduces ER stress in vivo, restores mitochondrial complex-I activity, reduces dopaminergic neuron loss, and improves behavioral symptoms in *Drosophila* and rodent models [185, 260, 261]. A clinical trial of ambroxol (ClinicalTrials.gov ID: NCT02941822) has demonstrated good cerebrospinal fluid penetration, safety, and tolerability while improving motor scores in patients [253]. These encouraging results paved the way for a phase 3 trial of ambroxol for the treatment of *GBA1*-PD, which will commence enrollment in the UK.

### *GBA1*-targeting therapy

**Fig. 6:**
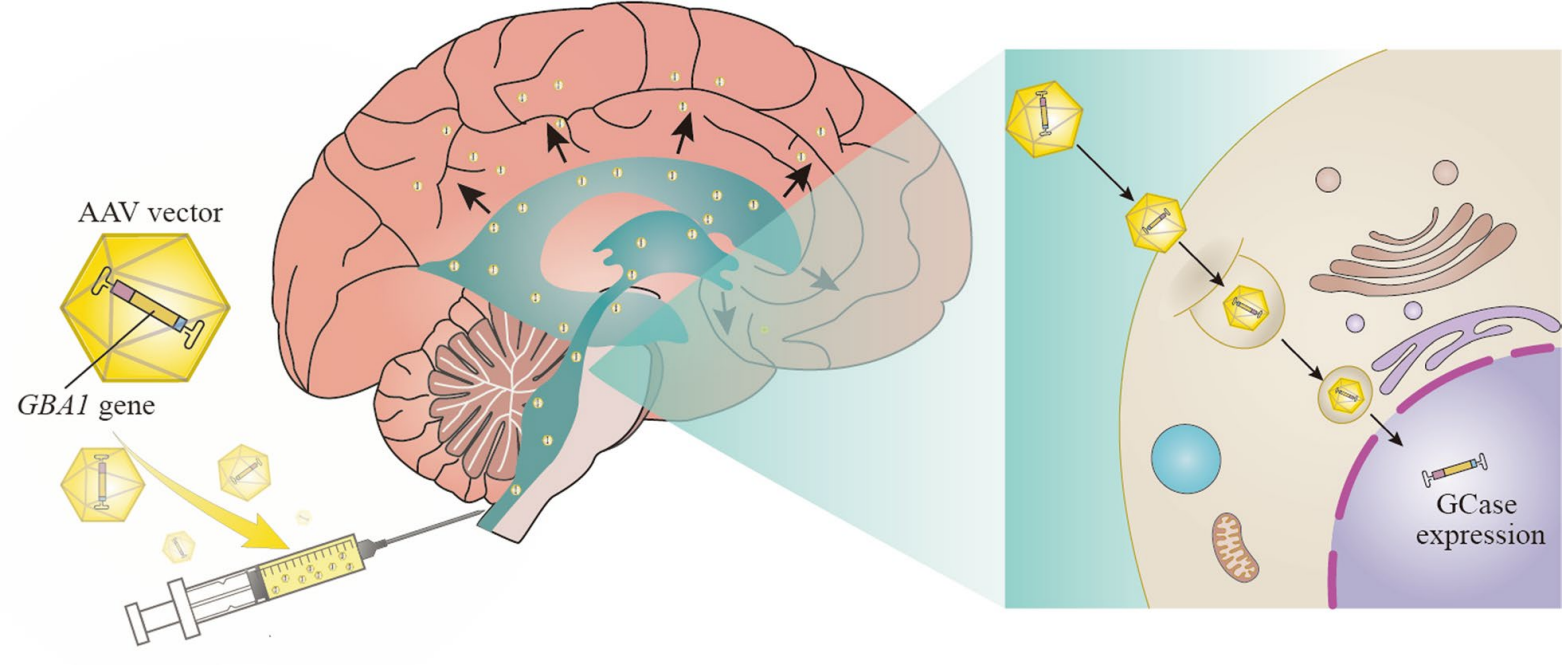
Gene therapies for *GBA1*-PD. Using the adeno-associated virus (AAV) as a vector, the normal *GBA1* gene is introduced into the cells of patients through intracisternal injection to correct the defective gene and produce functional glucocerebrosidase (GCase) proteins, which in turn alleviates the disease

Gene therapies entail rectifying disease-related genetic abnormalities by introducing therapeutic genes and their accompanying regulatory components into the cell nucleus. In the context of *GBA1*-PD, gene therapies often utilize viral vectors carrying intact *GBA1* gene sequences (Fig. 6). Direct administration or systemic injection of a functioning *GBA1* gene into animals increased GCase activity, decreased GlcSph accumulation, and reduced α-syn accumulation, thereby improving motor and memory deficits and protecting dopaminergic neurons [24, 262]. Based on this promising evidence, a clinical trial of AAV9 (adeno-associated virus 9)-based gene therapy (LY3884961, formerly known as PR001) is currently underway for *GBA1-*PD. This phase 1/2a clinical trial involves administering multiple dosages of LY3884961 to evaluate its safety, tolerability, immunogenicity, biomarkers, and clinical effectiveness.

Since there are different classifications of *GBA1* variants, pharmacological studies targeting *GBA1*-PD should carefully consider the differences between the various *GBA1* variants to ensure that treatment is consistent with the mechanism of mutation and specificity of the *GBA1* variant. Different *GBA1* variants may result in diverse structures and functions of GCase. Certain variants may induce ER retention, preventing GCase from reaching the lysosome or causing ER stress and cellular demise [15, 184]; truncating mutations result in protein deletion and thus loss of function [23]; and others may affect enzyme function by altering the active site conformation [263]. Therefore, a balanced distribution of different mutations is essential during selection of participants to minimize the mutation-related bias. In addition, close attention should be paid to GCase modifiers such as *CTSB*, *TMEM175*, and *LRRK2* when enrolling participants [65, 170, 237].

## Conclusions

There is growing evidence underscoring the importance of *GBA1* mutations in PD etiology. Research investigating the genotype-phenotype characteristics, pathogenesis, biomarkers, and targeted treatments for *GBA1*-PD has yielded significant insights. However, current understanding of the biological effects of various mutations remains incomplete. It is difficult to fully unravel the complexity of neurodegenerative diseases in cellular and animal models, so it is crucial to select appropriate *GBA1*-PD models according to the research objectives. It is believed that with the development of iPSCs, midbrain organoids, endolysosomal models, genome editing, optogenetics, and other technologies, more complete technical support will be provided to explore the mechanism of *GBA1*-PD [190, 213, 217, 264–266]. The treatment of *GBA1*-PD should be individualized, taking into account the *GBA1* mutation status, other genetic factors, and comprehensive clinical features [244]. Because each therapeutic strategy has specific strengths and limitations, it is necessary to combine multiple therapeutic strategies to combat PD [267]. It is believed that with the emergence of novel therapies and technologies, such as GCase modulators, cell treatment, antisense oligonucleotides, microRNAs, ncRNAs, and CRISPR-Cas9, among others, there will be new opportunities for the treatment of *GBA1*-PD [38, 107, 110, 252, 268–271]. Finally, with the accumulation of cohort data, the establishment of standardized experimental procedures, the development of global cooperation, the advancement of detection technology, and the improvement of diagnosis and treatment, advances will be made in the clinical phenotypes, etiological mechanisms, biomarkers, and interventions and treatments for *GBA1*-PD.

## Supplementary Information

**Additional file 1: Table S1** *GBA1* variants reported in PD.

## Data Availability

Not applicable.

## Abbreviations

α-syn: Alpha-synuclein
AD: Alzheimer’s disease
ALP: Autophagy-lysosomal pathway
BBB: Blood–brain barrier
*BIN1*: Bridging integrator 1
CBE: Conduritol B epoxide
CMA: Chaperone-mediated autophagy
DLB: Dementia with Lewy bodies
ER: Endoplasmic reticulum
ERT: Enzyme replacement therapy
*GBA1*: Glucocerebrosidase gene
GCase: Glucocerebrosidase
GD: Gaucher’s disease
GlcCer: Glucosylceramide
GlcSph: Glucosylsphingosine
GSLs: Glycosphingolipids
IL: Interleukin
LBs: Lewy bodies
*LRRK2*: Leucine-rich repeat kinase 2
LSD: Lysosome storage disorder
PBMC: Peripheral blood mononuclear cell
PD: Parkinson’s disease
RBD: Rapid eye movement sleep behavior disorder
ROS: Reactive oxygen species
SRT: Substrate reduction therapy
UPR: Unfolded protein response
