# Supplementary material for: Clinical, mechanistic, biomarker, and therapeutic advances in GBA1-associated Parkinson’s disease
Source: Transl Neurodegener. 2024 Sep 12;13:48. doi: 10.1186/s40035-024-00437-6 (PMC11391654; doi:10.1186/s40035-024-00437-6)
Supplement: Supplementary file 1 — Additional file 1: Table S1 GBA1 variants reported in PD. [file 40035_2024_437_MOESM1_ESM.docx]

**Table S1** GBA1 variants reported in PD.

| **Variant Name** | **cDNA** | **Full length protein** | **Variant type (Severe, Mild, Risk Factor)** | **ACMG Classification of variant in Gaucher Disease** |
| --- | --- | --- | --- | --- |
| c.-30G>C | c.-30G>C | n/a | Unknown | Uncertain significance |
| c.-22T>C | c.-22T>C | n/a | Unknown | Uncertain significance |
| c.-2G>T | c.-2G>T | n/a | Unknown | Uncertain significance |
| p.R(-32)T | c.23G>C | p.(Arg8Thr) | Unknown | Uncertain significance |
| p.E(-31)Gfs*8 | c.26_27del | p.(Glu9GlyfsTer8) | Severe (Null) | Pathogenic |
| p.E(-30)* | c.28G>T | p.(Glu10Ter) | Severe (Null) | Pathogenic |
| IVS1+1G>T | c.27+1G>T | n/a | Unknown | Uncertain significance |
| IVS1+191G>C | c.27+191G>C | n/a | Unknown | Uncertain significance |
| IVS1-16C>G | c.28-16C>G | n/a | Unknown | Uncertain significance |
| IVS1-14T>A | c.28-14T>A | n/a | Unknown | Uncertain significance |
| p.P(-28)S | c.34C>T | p.(Pro12Ser) | Unknown | Uncertain significance |
| p.K(-27)R | c.38A>G | p.(Lys13Arg) | Unknown | Likely benign |
| p.P(-26)T | c.40C>A | p.(Pro14Thr) | Unknown | Uncertain significance |
| p.P(-26)P | c.42T>G | p.(Pro14Pro) | Unknown | Likely benign |
| p.L(-25)S | c.44T>C | p.(Leu15Ser) | Unknown | Uncertain significance |
| p.L(-25)S + p.S(-24)G | c.[44T>C;46A>G] | p.[(Leu15Ser;Ser16Gly)] | Unknown + Unknown | Uncertain significance + Uncertain significance |
| p.S(-24)G | c.46A>G | p.(Ser16Gly) | Unknown | Uncertain significance |
| p.I(-20)V | c.58A>G | p.(Ile20Val) | Unknown | Likely benign |
| p.L(-15)V | C.73C>G | p.(Leu25Val) | Unknown | Uncertain significance |
| p.L(-11)Afs*18 | c.84dupG | p.(Leu29AlafsTer18) | Severe (Null) | Pathogenic |
| p.Q(-8)R | c.95A>G | p.(Gln32Arg) | Unknown | Uncertain significance |
| p.A(-7)V | c.98C>T | p.(Ala33Val) | Unknown | Uncertain significance |
| p.V(-6)Cfs*56 | c.100delG | p.(Val34Cysfs*56) | Severe (Null) | Pathogenic |
| p.W(-4)* | c.108G>A | p.(Trp36Ter) | Severe (Null) | Pathogenic |
| p.S(-2)T | c.112T>A | p.(Ser38Thr) | Unknown | Uncertain significance |
| p.G(-1)R | c.115G>C | p.(Gly39Arg) | Unknown | Uncertain significance |
| IVS2+1G>T | c.115+1G>T | - | Severe | Uncertain significance |
| IVS2+1G>A | c.115+1G>A | - | Severe (Null) | Pathogenic |
| IVS2-8C>T | c.116-8C>T | - | Unknown | Uncertain significance |
| p.P3A | c.124C>G | p.(Pro42Ala) | Unknown | Uncertain significance |
| p.P3P | c.126C>T | p.(Pro42Pro) | Unknown | Likely benign |
| p.K7E | c.136A>G | p.(Lys46Glu) | Unknown | Uncertain significance |
| p.G10S | c.145G>A | p.(Gly49Ser) | Unknown | Uncertain significance |
| p.Y11H | c.148T>C | p.(Tyr50His) | Unknown | Uncertain significance |
| p.S13L | c.155C>T | p.(Ser52Leu) | Unknown | Uncertain significance |
| p.S13S | c.156G>A | p.(Ser52Ser) | Unknown | Likely benign |
| p.V14Wfs*37 | c.157delG | p.(Val53TrpfsTer37) | Severe (Null) | Pathogenic |
| p.V17Lfs*6 | c.166_167del | p.(Val56LeufsTer6) | Severe (Null) | Pathogenic |
| p.V17V | c.168C>T | p.(Val56Val) | Unknown | Uncertain significance |
| p.C18S | c.170G>C | p.(Cys57Ser) | Unknown | Uncertain significance |
| p.C18* | c.171C>A | p.(Cys57Ter) | Severe (Null) | Pathogenic |
| p.N19S | c.173A>G | p.(Asn58Ser) | Unknown | Uncertain significance |
| p.D24N | c.187G>A | p.(Asp63Asn) | Severe | Uncertain significance |
| p.P29Rfs*22 | c.203delC | p.(Pro68ArgfsTer22) | Severe (Null) | Pathogenic |
| p.T30Dfs*11 | c.203dupC | p.(Thr69Aspfs*11) | Severe (Null) | Pathogenic |
| p.P32L | c.212C>T | p.(Pro71Leu) | Unknown | Uncertain significance |
| p.G35A | c.221G>C | p.(Gly74Ala) | Unknown | Uncertain significance |
| p.T36del | c.222_224delTAC | p.(Thr75del) | Unknown | No data |
| p.F37V | c.226T>G | p.(Phe76Val) | Mild | Uncertain significance |
| p.S38G | c.229A>G | p.(Ser77Gly) | Unknown | Uncertain significance |
| p.R39C | c.232C>T | p.(Arg78Cys) | Unknown | Uncertain significance |
| p.R39H | c.233G>A | p.(Arg78His) | Unknown | Uncertain significance |
| p.R44C | c.247C>T | p.(Arg83Cys) | Unknown | Uncertain significance |
| p.S45Rfs*15 | c.251_252insC | p.(Ser84ArgfsTer15) | Severe (Null) | Pathogenic |
| p.G46E | c.254G>A | p.(Gly85Glu) | Mild | Pathogenic |
| p.R47* | c.256C>T | p.(Arg86Ter) | Severe (Null) | Pathogenic |
| p.R48W | c.259C>T | p.(Arg87Trp) | Mild | Pathogenic |
| p.P55S | c.280C>T | p.(Pro94Ser) | Unknown | Uncertain significance |
| p.I56V | c.283A>G | p.(Ile95Val) | Unknown | Uncertain significance |
| p.N59Kfs*47 | c.293dupA | p.(Asn98LysfsTer47) | Severe (Null) | Pathogenic |
| p.T61M | c.299C>T | p.(Thr100Met) | Unknown | Uncertain significance |
| p.T61T | c.300G>A | p.(Thr100Thr) | Unknown | Uncertain significance |
| IVS3+1G>T | c.307+1G>T | - | Severe | Pathogenic |
| IVS3+1G>A | c.307+1G>A | - | Severe | Pathogenic |
| IVS3-1G>C | c.308-1G>A | - | Severe | Pathogenic |
| p.G64V | c.308G>T | p.(Gly103Val) | Unknown | Uncertain significance |
| p.L66P | c.314T>C | p.(Leu105Pro) | Mild | Uncertain significance |
| p.L66Q | c.314T>A | p.(Leu105Gln) | Unknown | Uncertain significance |
| del5 | c.334_338delCAGAA | p.(Lys114ValfsTer31) | Severe (Null) | Pathogenic |
| p.V79A | c.350T>C | p.(Val118Ala) | Unknown | Uncertain significance |
| p.G80R | c.355G>A | p.(Gly119Arg) | Unknown | Uncertain significance |
| p.M85V | c.370A>G | p.(Met124Val) | Unknown | Uncertain significance |
| p.M85T | c.371T>C | p.(Met124Thr) | Unknown | Uncertain significance |
| p.A95S | c.400G>T | p.(Ala134Ser) | Unknown | Uncertain significance |
| p.L105I | c.430C>A | p.(Leu144Ile) | Unknown | Uncertain significance |
| p.S107L | c.437C>T | p.(Ser146Leu) | Severe | Pathogenic |
| p.Y108C | c.440A>G | p.(Tyr147Cys) | Unknown | Uncertain significance |
| IVS4+2T>A | c.454+2T>A | - | Severe | Pathogenic |
| IVS4+47G>A | c.454+47G>A | - | Unknown | Benign |
| IVS4-2A>G + c.(-203)A>G | c.[455-2A>G;(-203)A>G] | - | Severe | Pathogenic |
| p.G113A | c.455G>C | p.(Gly152Ala) | Unknown | Uncertain significance |
| p.I119I | c.474C>T | p.(Ile158Ile) | Unknown | Uncertain significance |
| p.R120W | c.475C>T | p.(Arg159Trp) | Severe | Pathogenic |
| p.R120Q | c.476G>A | p.(Arg159Gln) | Severe | Likely pathogenic |
| p.R120W/p.E388K | c.[475C>T];[1279G>A] | p.[(Arg159Trp)];[(Glu427Lys)] | Severe/Risk Variant | Pathogenic/Uncertain significance |
| p.V121V | c.480A>T | p.(Val160Val) | Unknown | Likely benign |
| p.P122L | c.482C>T | p.(Pro161Leu) | Severe | Uncertain significance |
| p.P122R | c.482C>G | p.(Pro161Arg) | Unknown | Uncertain significance |
| p.M123V | c.484A>G | p.(Met162Val) | Mild | Uncertain significance |
| p.M123T | c.485T>C | p.(Met162Thr) | Unknown | Uncertain significance |
| p.A124T | c.487G>A | p.(Ala163Thr) | Unknown | Uncertain significance |
| p.S125N | c.491G>A | p.(Ser164Asn) | Unknown | Uncertain significance |
| p.S125R | c.492C>G | p.(Ser164Arg) | Unknown | Likely pathogenic |
| p.D127E | c.498C>G | p.(Asp166Glu) | Unknown | Uncertain significance |
| p.F128Ffs*12 | c.500insT | p.(Phe167PhefsTer12) | Severe (Null) | Pathogenic |
| p.I130S | c.506T>G | p.(Ile169Ser) | Unknown | Uncertain significance |
| p.I130I | c.507C>A | p.(Ile169Ile) | Unknown | Uncertain significance |
| p.R131C | c.508C>T | p.(Arg170Cys) | Severe | Pathogenic |
| p.R131L | c.509G>T | p.(Arg170Leu) | Severe | Likely pathogenic |
| p.R131H | c.509G>A | p.(Arg170His) | Unknown | Likely pathogenic |
| p.T134P | c.517A>C | p.(Thr173Pro) | Unknown | Uncertain significance |
| p.Y135C | c.521A>G | p.(Tyr174Cys) | Unknown | Uncertain significance |
| p.D140H | c.535G>C | p.(Asp179His) | Unknown | Likely pathogenic |
| p.D140H + p.E326K | c.[535G>C;1093G>A] | p.[(Asp179His;Glu365Lys)] | Mild + Risk variant | Likely pathogenic + Benign |
| p.D140H + p.E326K / p.T369M | c.[535G>C;1093G>A];[1223C>T] | p.[(Asp179His;Glu365Lys)];[(Thr408Met)] | Mild + Risk variant/Risk variant | Likely pathogenic + Benign/Uncertain significance |
| p.D140H + p.E326K / p.E326K | c.[535G>C;1093G>A];[1093G>A] | p.[(Asp179His;Glu365Lys)];[(Glu365Lys)] | Mild + Risk variant/Risk variant | Likely pathogenic + Benign/Benign |
| p.Q143* | c.544C>T | p.(Gln182Ter) | Severe (Null) | Likely pathogenic |
| p.Q143Q | c.546G>A | p.(Gln182Gln) | Unknown | Likely benign |
| p.L144V | c.547T>G | p.(Leu183Val) | Unknown | Uncertain significance |
| p.K157Q | c.586A>C | p.(Lys196Gln) | Severe | Likely pathogenic |
| IVS5+7A>C | c.588+7A>C | - | Unknown | Uncertain significance |
| IVS5-86A>G | c.589-86A>G | - | Unknown | Benign |
| p.I161N | c.599T>A | p.(Ile200Asn) | Unknown | Uncertain significance |
| p.R163* | c.604C>T | p.(Arg202Ter) | Severe (Null) | Pathogenic |
| p.R163Q | c.605G>A | p.(Arg202Gln) | Unknown | Uncertain significance |
| p.R163Q + p.L444P | c.[605G>A;c.1448T>C] | p.[(Arg202Gln;Leu483Pro)] | Unknown + Severe | Uncertain significance + Pathogenic |
| p.R163Q + p.V460M | c.[605G>A;1495G>A] | p.[(Arg202Gln;Val499Met)] | Unknown + Unknown | Uncertain significance + Likely pathogenic |
| p.R170H | c.626G>A | p.(Arg209His) | Unknown | Uncertain significance |
| p.V172L | c.631G>C | p.(Val211Leu) | Unknown | Uncertain significance |
| p.S173* | c.635C>G | p.(Ser212Ter) | Severe (Null) | Pathogenic |
| p.S173S | c.636A>C | p.(Ser212Ser) | Unknown | Likely benign |
| p.L174P | c.638T>C | p.(Leu213Pro) | Unknown | Uncertain significance |
| p.L175I | c.640C>A | p.(Leu214Ile) | Unknown | Uncertain significance |
| p.S177T + p.V172L | c.647G>C(;)631G>C | p.(Ser216Thr)(;)(Val211Leu) | Unknown;Unknown | Uncertain significance;Uncertain significance |
| p.P182L | c.662C>T | p.(Pro221Leu) | Severe | Uncertain significance |
| p.W184R | c.667T>C | p.(Trp223Arg) | Severe | Pathogenic |
| p.L185R | c.671T>G | p.(Leu224Arg) | Unknown | Uncertain significance |
| p.K186N | c.675G>C | p.(Lys225Asn) | Unknown | Uncertain significance |
| p.N188S | c.680A>G | p.(Asn227Ser) | Severe | Pathogenic |
| p.N188K | c.681T>G | p.(Asn227Lys) | Severe | Likely pathogenic |
| p.N188K + p.W184R | c.681T>G(;)667T>C | p.(Asn227Lys)(;)(Trp223Arg) | Severe;Severe | Likely pathogenic;Pathogenic |
| p.G189V | c.683G>T | p.(Gly228Val) | Mild | Uncertain significance |
| p.A190T | c.685G>A | p.(Ala229Thr) | Severe | Uncertain significance |
| p.N192K | c.693T>A | p.(Asn231Lys) | Unknown | Uncertain significance |
| p.G193R | c.694G>A | p.(Gly232Arg) | Mild | Uncertain significance |
| p.G193W | c.694G>T | p.(Gly232Trp) | Severe | Uncertain significance |
| p.G193E | c.695G>A | p.(Gly232Glu) | Unknown | Uncertain significance |
| p.G193G | c.696G>A | p.(Gly232Gly) | Unknown | Likely benign |
| p.G195W | c.700G>T | p.(Gly234Trp) | Severe | Uncertain significance |
| p.G195E | c.701G>A | p.(Gly234Glu) | Unknown | Likely pathogenic |
| p.G195G | c.702G>T | p.(Gly234Gly) | Unknown | Likely benign |
| p.S196P | c.703T>C | p.(Ser235Pro) | Unknown | Pathogenic |
| p.S196P + p.G202R | c.[703T>C;721G>A] | p.[(Ser235Pro;Gly241Arg)] | Unknown + Severe | Pathogenic + Pathogenic |
| p.L197F | c.706C>T | p.(Leu236Phe) | Severe | Uncertain significance |
| p.L197P | c.707T>C | p.(Leu236Pro) | Unknown | Uncertain significance |
| p.L197Lfs*17 | c.708delC | p.(Leu236LeufsTer17) | Severe (Null) | Pathogenic |
| p.K198E | c.709A>G | p.(Lys237Glu) | Severe | Pathogenic |
| p.G199R | c.712G>A | p.(Gly238Arg) | Unknown | Uncertain significance |
| p.P201H | c.719C>A | p.(Pro240His) | Severe | Uncertain significance |
| p.G202R | c.721G>A | p.(Gly241Arg) | Severe | Pathogenic |
| p.G202R + p.S196P + p.V191G + p.N188K + p.N188S | c.[721G>A;703T>C;689T>G;681T>G;680A>G] | p.[(Gly241Arg;Ser235Pro;Val230Gly;Asn227Lys;Asn227Ser)] | Severe + Unknown + Unknown + Severe + Severe | Pathogenic + Pathogenic + Uncertain significance + Likely pathogenic + Pathogenic |
| p.G202R + p.E326K | c.721G>A(;)1093G>A | p.(Gly241Arg)(;)(Glu365Lys) | Severe;Risk variant | Pathogenic;Benign |
| p.Y205C | c.731A>G | p.(Tyr244Cys) | Mild | Uncertain significance |
| p.T208S | c.740C>G | p.(Thr247Ser) | Unknown | Uncertain significance |
| p.T208I | c.740C>T | p.(Thr247Ile) | Unknown | Uncertain significance |
| p.T208T | c.741C>T | p.(Thr247Thr) | Unknown | Likely benign |
| p.W209Gfs*6 | c.741delC | p.(Trp248GlyfsTer6) | Severe (Null) | Pathogenic |
| p.W209* | c.744G>A | p.(Trp248Ter) | Severe (Null) | Pathogenic |
| p.R211K | c.749G>A | p.(Arg250Lys) | Unknown | Uncertain significance |
| p.F213I | c.754T>A | p.(Phe252Ile) | Severe | Pathogenic |
| IVS6-86A>G | c.762-86A>G | - | Unknown | Uncertain significance |
| IVS6-18T>A | c.762-18T>A | - | Unknown | Uncertain significance |
| IVS6-5G>A | c.762-5G>A | - | Unknown | Uncertain significance |
| IVS6-2A>G | c.762-2A>G | - | Severe | Pathogenic |
| IVS6-1G>C | c.762-1G>C | - | Severe | Pathogenic |
| p.F216Y | c.764T>A | p.(Phe255Tyr) | Mild | Pathogenic |
| p.F216L | c.765C>(A or G) | p.(Phe255Leu) | Unknown | Uncertain significance |
| p.L217P | c.767T>C | p.(Leu256Pro) | Unknown | Uncertain significance |
| p.Y220C | c.776A>G | p.(Tyr259Cys) | Unknown | Uncertain significance |
| p.W228* | c.801G>A | p.(Trp267Ter) | Severe (Null) | Pathogenic |
| p.E239K | c.832G>A | p.(Glu278Lys) | Unknown | Benign |
| p.P245L | c.851C>T | p.(Pro284Leu) | Unknown | Uncertain significance |
| p.C248R | c.859T>C | p.(Cys287Arg) | Unknown | Uncertain significance |
| p.G250S | c.865G>A | p.(Gly289Ser) | Unknown | Uncertain significance |
| p.G250V | c.866G>T | p.(Gly289Val) | Unknown | Likely pathogenic |
| p.E254Nfs*10 | c.876delT | p.(Glu293AsnfsTer10) | Severe (Null) | Pathogenic |
| p.H255Q | c.882T>G | p.(His294Gln) | Severe | Pathogenic |
| p.H255Q + p.T369M | c.882T>G(;)1223C>T | p.(His294Gln)(;)(Thr408Met) | Severe;Risk variant | Pathogenic;Uncertain significance |
| p.H255Q + p.D409H | c.[882T>G;1342G>C] | p.[(His294Gln;Asp448His)] | Severe + Severe | Pathogenic + Pathogenic |
| p.H255Q + p.D409H | c.882T>G(;)1342G>C | p.(His294Gln)(;)(Asp448His) | Severe;Severe | Pathogenic;Pathogenic |
| p.Q256Sfs*9 | c.883delC | p.(Gln295SerfsTer9) | Severe (Null) | Pathogenic |
| p.R257* | c.886C>T | p.(Arg296Ter) | Severe (Null) | Pathogenic |
| p.R257Q | c.887G>A | p.(Arg296Gln) | Severe | Pathogenic |
| p.I260T | c.896T>C | p.(Ile299Thr) | Unknown | Likely pathogenic |
| p.R262C | c.901C>T | p.(Arg301Cys) | Unknown | Uncertain significance |
| p.R262H | c.902G>A | p.(Arg301His) | Unknown | Uncertain significance |
| p.R262P | c.902G>C | p.(Arg301Pro) | Unknown | Uncertain significance |
| p.L264V | c.907C>G | p.(Leu303Val) | Unknown | Uncertain significance |
| p.L264I | c.907C>A | p.(Leu303Ile) | Mild | Uncertain significance |
| p.G265S | c.910G>A | p.(Gly304Ser) | Unknown | Uncertain significance |
| p.P266Lfs*31 | c.914delC | p.(Pro305LeufsTer31) | Severe (Null) | Pathogenic |
| p.T267I | c.917C>T | p.(Thr306Ile) | Unknown | Uncertain significance |
| p.L268L | c.921C>T | p.(Leu307Leu) | Unknown | Uncertain significance |
| p.A269T | c.922G>A | p.(Ala308Thr) | Unknown | Uncertain significance |
| p.S271G | c.928A>G | p.(Ser310Gly) | Mild | Likely pathogenic |
| p.S271N | c.929G>A | P.(Ser310Asn) | Mild | Likely pathogenic |
| p.S271T | c.929G>C | p.(Ser310Thr) | Unknown | Likely pathogenic |
| p.H274R | c.938A>G | p.(His313Arg) | Unknown | Uncertain significance |
| p.V276F | c.943G>T | p.(Val315Phe) | Unknown | Uncertain significance |
| p.R277C | c.946C>T | p.(Arg316Cys) | Mild | Uncertain significance |
| p.R277H | c.947G>A | p.(Arg316His) | Unknown | Uncertain significance |
| p.L279Pfs*17 | c.953delT | p.(Leu318ProfsTer17) | Severe (Null) | Pathogenic |
| p.R285H | c.971G>A | p.(Arg324His) | Severe | Uncertain significance |
| p.P289Rfs*11 | c.983_990del | p.(Pro328ArgfsTer11) | Severe (Null) | Pathogenic |
| p.W291R | c.988T>A | p.(Trp330Arg) | Unknown | Uncertain significance |
| IVS7-4G>T | c.1000-4G>T | - | Unknown | Uncertain significance |
| p.P299T | c.1012C>A | p.(Pro338Thr) | Unknown | Uncertain significance |
| p.A301T | c.1018G>A | p.(Ala340Thr) | Unknown | Uncertain significance |
| p.Y304C | c.1028A>G | p.(Tyr343Cys) | Severe | Uncertain significance |
| p.I308T | c.1040T>C | p.(Ile347Thr) | Unknown | Uncertain significance |
| p.H311Y | c.1048C>T | p.(His350Tyr) | Unknown | Uncertain significance |
| p.H311R | c.1049A>G | p.(His350Arg) | Severe | Likely pathogenic |
| p.W312R | c.1051T>A | p.(Trp351Arg) | Unknown | Uncertain significance |
| p.Y313H | c.1054T>C | p.(Tyr352His) | Unknown | Uncertain significance |
| p.L314V | c.1057C>G | p.(Leu353Val) | Unknown | Uncertain significance |
| p.F316I | c.1063T>A | p.(Phe355Ile) | Unknown | Uncertain significance |
| p.L317V | c.1066C>G | p.(Leu356Val) | Unknown | Uncertain significance |
| p.L317L | c.1068G>A | p.(Leu356Leu) | Unknown | Likely benign |
| p.K321R | c.1079A>G | p.(Lys360Arg) | Unknown | Uncertain significance |
| p.T323I | c.1085C>T | p.(Thr362Ile) | Severe | Pathogenic |
| p.L324V | c.1087C>G | p.(Leu363Val) | Unknown | Uncertain significance |
| p.L324I | c.1087C>A | p.(Leu363Ile) | Unknown | Uncertain significance |
| p.L324P | c.1088T>C | p.(Leu363Pro) | Unknown | Uncertain significance |
| p.L324P / p.T369M | c.[1088T>C];[1223C>T] | p.[(Leu363Pro)];[(Thr408Met)] | Unknown/Risk variant | Uncertain significance/Uncertain significance |
| p.G325R | c.1090G>A | p.(Gly364Arg) | Severe | Likely pathogenic |
| p.G325W | C.1090G>T | p.(Gly364Trp) | Unknown | Likely pathogenic |
| p.E326K | c.1093G>A | p.(Glu365Lys) | Risk variant | Benign |
| p.E326K / p.G64D | c.[1093G>A];[308G>A] | p.[(Glu365Lys)];[(Gly103Asp)] | Risk variant/Unknown | Benign/Uncertain significance |
| p.E326K + p.N188S | c.[1093G>A;680A>G] | p.[(Glu365Lys;Asn227Ser)] | Risk variant + Severe | Benign + Pathogenic |
| p.E326K / p.E326K | c.[1093G>A];[1093G>A] | p.[(Glu365Lys)];[(Glu365Lys)] | Risk variant/Risk variant | Benign/Benign |
| p.E326K / p.T369M | c.[1093G>A];[1223C>T] | p.[(Glu365Lys)];[(Thr408Met)] | Risk variant/Risk variant | Benign/Uncertain significance |
| p.E326K / p.N370S | c.[1093G>A];[1226A>G] | p.[(Glu365Lys)];[(Asn409Ser)] | Risk variant/Mild | Benign/Likely pathogenic |
| p.E326K + p.L444P | c.1093G>A(;)1448T>C | p.(Glu365Lys)(;)(Leu483Pro) | Risk variant;Severe | Benign;Pathogenic |
| p.E326D | c.1095G>C | p.(Glu365Asp) | Unknown | Uncertain significance |
| p.H328Tfs*68 | c.1098insA | p.(His367ThrfsTer68) | Severe (Null) | Pathogenic |
| p.R329C | c.1102C>T | p.(Arg368Cys) | Mild | Likely pathogenic |
| p.R329G | c.1102C>G | p.(Arg368Gly) | Unknown | Uncertain significance |
| p.R329H | c.1103G>A | p.(Arg368His) | Unknown | Uncertain significance |
| p.L336L | c.1125C>(A,T, or G) | p.(Leu375Leu) | Unknown | Uncertain significance |
| p.S339L | c.1133C>T | p.(Ser378Leu) | Unknown | Uncertain significance |
| p.E340A | c.1136A>C | p.(Glu379Ala) | Unknown | Uncertain significance |
| p.C342F | c.1142G>T | p.(Cys381Phe) | Unknown | Uncertain significance |
| p.C381_V382del | c.1143_1145del | p.(Cys381_Val382del) | Severe (Null) | Pathogenic |
| p.G344S | c.1147G>A | p.(Gly383Ser) | Unknown | Uncertain significance |
| p.G344G | c.1149C>T | p.(Gly383Gly) | Unknown | Likely benign |
| p.F347L | c.1156T>C | p.(Phe386Leu) | Unknown | Uncertain significance |
| p.W348G | c.1159T>G | p.(Trp387Gly) | Unknown | Uncertain significance |
| p.Q350H | c.1167G>C | p.(Gln389His) | Unknown | Uncertain significance |
| p.V352G | c.1172T>G | p.(Val391Gly) | Unknown | Uncertain significance |
| p.R353W | c.1174C>T | p.(Arg392Trp) | Unknown | Pathogenic |
| p.R353Gfs*1 | c.1174delC | p.(Arg392GlyfsTer*1) | Severe (Null) | Pathogenic |
| p.L354P | c.1178T>C | p.(Leu393Pro) | Unknown | Uncertain significance |
| p.W357R | c.1186T>C | p.(Trp396Arg) | Unknown | Uncertain significance |
| p.R359* | c.1192C>T | p.(Arg398Ter) | Severe (Null) | Pathogenic |
| p.M361Lfs*2 | c.1197_1198insCTGTA | p.(Met400LeufsTer2) | Severe (Null) | Pathogenic |
| p.M361I | c.1200G>A | p.(Met400Ile) | Unknown | Uncertain significance |
| p.Q362* | c.1201C>T | p.(Gln401Ter) | Unknown | Pathogenic |
| p.S364R | c.1207A>C | p.(Ser403Arg) | Severe | Uncertain significance |
| p.S364N | c.1208G>A | p.(Ser403Asn) | Unknown | Likely pathogenic |
| p.S364S | c.1209C>T | p.(Ser403Ser) | Unknown | Likely benign |
| p.I367N | c.1217T>A | p.(Ile406Asn) | Unknown | Uncertain significance |
| p.I368N | c.1220T>A | p.(Ile407Asn) | Unknown | Uncertain significance |
| p.I368T | c.1220T>C | p.(Ile407Thr) | Unknown | Uncertain significance |
| p.T369M | c.1223C>T | p.(Thr408Met) | Risk Variant | Uncertain significance |
| p.T369M / p.T369M | c.[1223C>T];[1223C>T] | p.[(Thr408Met)];[(Thr408Met)] | Risk Variant/Risk Variant | Uncertain significance/Uncertain significance |
| p.T369M + p.D409H | c.[1223C>T;1342G>C] | p.[(Thr408Met);(Asp448His)] | Risk variant + Severe | Uncertain significance + Pathogenic |
| p.T369M + IVS10-12C>T | c.1223C>T(;)1506-12C>T | p.(Thr408Met) | Risk variant;Unknown | Uncertain significance;Uncertain significance |
| p.T369T | c.1224G>A | p.(Thr408Thr) | Unknown | Uncertain significance |
| IVS8-34C>A | c.1225-34C>A | - | Unknown | Uncertain significance |
| IVS8-24T>G | c.1225-24T>G | - | Unknown | Uncertain significance |
| p.N370S | c.1226A>G | p.(Asn409Ser) | Mild | Likely pathogenic |
| p.N370S + p.E326K | c.1226A>G(;)1093G>A | p.(Asn409Ser)(;)(Glu365Lys) | Mild;Risk variant | Likely pathogenic;Benign |
| p.N370S / p.G377S | c.[1226A>G];[1246G>A] | p.[(Asn409Ser)];[(Gly416Ser)] | Mild/Severe | Likely pathogenic/Pathogenic |
| p.N370S / p.P401L | c.[1226A>G];[(1319C>T)] | p.[(Asn409Ser)];[(Pro440Leu)] | Mild/Unknown | Likely pathogenic/Likely pathogenic |
| p.N370S / p.D409H + p.H255Q | c.[1226A>G];[1342G>C;c.882T>G] | p.[(Asn409Ser)];[(Asp448His);(His294Gln)] | Mild/Severe + Severe | Likely pathogenic/Pathogenic + Pathogenic |
| p.N370S / p.G454D | c.[1226A>G];[1478G>A] | p.[(Asn409Ser)];[(Gly454Asp)] | Mild/Unknown | Likely pathogenic/Uncertain significance |
| p.L372P | c.1232T>C | p.(Leu411Pro) | Unknown | Uncertain significance |
| p.V375L | c.1240G>T | p.(Val414Leu) | Mild | Pathogenic/Likely pathogenic |
| p.V375G | c.1241T>G | p.(Val414Gly) | Unknown | Uncertain significance |
| p.V376V | c.1245C>T | p.(Val415Val) | Unknown | Likely benign |
| p.G377C | c.1246G>T | p.(Gly416Cys) | Mild | Likely pathogenic |
| p.G377S | c.1246G>A | p.(Gly416Ser) | Severe | Pathogenic |
| p.G377D | c.1247G>A | p.(Gly416Asp) | Unknown | Uncertain significance |
| p.W378R | c.1249T>C | p.(Trp417Arg) | Unknown | Uncertain significance |
| p.W378G | c.1249T>G | p.(Trp417Gly) | Unknown | Likely pathogenic |
| p.W378T | c.1249_1251delTGC | p.(Trp417Thr) | Unknown | Uncertain significance |
| p.T379I | c.1253C>T | p.(Thr418Ile) | Unknown | Uncertain significance |
| p.D380N | c.1255G>A | p.(Asp419Asn) | Unknown | Uncertain significance |
| p.D380Y | c.1255G>T | p.(Asp419Tyr) | Unknown | Uncertain significance |
| p.D380A | c.1256A>C | p.(Asp419Ala) | Severe | Uncertain significance |
| del55 | ? | NA | Severe (Null) | Pathogenic |
| p.N382D | c.1261A>G | p.(Asn421Asp) | Unknown | Uncertain significance |
| p.N382K | c.1263C>A | p.(Asn421Lys) | Severe | Likely pathogenic |
| p.N382*fs | ? | p.(Asn421Terfs) | Severe (Null) | Pathogenic |
| p.L383Pfs*4 | c.1263_1317del55 | p.(Leu422ProfsTer4) | Severe (Null) | Pathogenic |
| p.L383Hfs*3 | c.1264_1319del55 | p.(Leu422HisfsTer3) | Severe (Null) | Pathogenic |
| p.L383Pfs*3 | c.1265_1319del | p.(Leu422ProfsTer3) | Severe (Null) | Pathogenic |
| P.L383*fs | ? | p.(Leu422Terfs) | Severe (Null) | Pathogenic |
| p.L383R | c.1265T>G | p.(Leu422Arg) | Unknown | Uncertain significance |
| p.A384V | c.1268C>T | p.(Ala423Val) | Unknown | Uncertain significance |
| p.A384D | c.1268C>A | p.(Ala423Asp) | Unknown | Uncertain significance |
| p.N386K | c.1275C>(A or G) | p.(Asn425Lys) | Unknown | Uncertain significance |
| p.P387L | c.1277C>T | p.(Pro426Leu) | Unknown | Uncertain significance |
| p.P387_E388insALNP | c.1279_1280insCCCTGAACCCCG | p.(Pro426_Glu427insAlaLeuAsnPro) | Unknown | Uncertain significance |
| p.E388K | c.1279G>A | p.(Glu427Lys) | Risk Variant | Uncertain significance |
| p.G390E | c.1286G>A | p.(Gly429Glu) | Unknown | Uncertain significance |
| p.G390V | c.1286G>T | p.(Gly429Val) | Unknown | Uncertain significance |
| p.P391L | c.1289C>T | p.(Pro430Leu) | Unknown | Pathogenic |
| p.N392S | c.1292A>G | p.(Asn431Ser) | Unknown | Uncertain significance |
| p.W393R | c.1294T>A | p.(Trp432Arg) | Unknown | Likely pathogenic |
| p.W393C | c.1296G>T | p.Trp432Cys | Unknown | Uncertain significance |
| p.W393* | c.1296G>A | p.(Trp432Ter) | Severe (Null) | Pathogenic |
| p.V394M | c.1297G>A | p.(Val433Met) | Unknown | Likely pathogenic |
| p.V394L | c.1297G>T | p.(Val433Leu) | Severe | Pathogenic |
| p.R395C | c.1300C>T | p.(Arg434Cys) | Unknown | Uncertain significance |
| p.N396T | c.1304A>C | p.(Asn435Thr) | Mild | Uncertain significance |
| p.V398Sfs*404 | c.1309delG | p.(Val437SerfsTer404) | Severe (Null) | Pathogenic |
| p.V398I | c.1309G>A | p.(Val437Ile) | Mild | Likely pathogenic |
| p.D399N | c.1312G>A | p.(Asp438Asn) | Severe | Pathogenic |
| p.D399H | c.1312G>C | p.(Asp438His) | Mild | Likely pathogenic |
| p.D409H | c.1342G>C | p.(Asp448His) | Severe | Pathogenic |
| RecTL | c.[1263-1317del;1342G>C;1448T>C;1483G>C;1497G>C] | p.[(Asp448His);(Leu483Pro);(Ala495Pro);(Val499Val)] | Severe | Pathogenic |
| p.T410M | c.1346C>T | p.(Thr449Met) | Unknown | Uncertain significance |
| p.T410T | c.1347G>C | p.(Thr449Thr) | Unknown | Likely benign |
| p.P415R | c.1361C>G | p.(Pro454Arg) | Severe | Pathogenic |
| p.H419Tfs*30 | c.1372delC | p.(His458ThrfsTer30) | Severe (Null) | Pathogenic |
| IVS9+1G>T | c.1388+1G>T | - | Severe | Pathogenic |
| IVS9+1G>A | c.1388+1G>A | - | Severe | Pathogenic |
| IVS9+3G>C | c.1388+3G>C | - | Unknown | Uncertain significance |
| IVS9+10T>G | c.1388+10T>G | - | Unknown | Uncertain significance |
| IVS9+32C>T | c.1388+32C>T | - | Unknown | Uncertain significance |
| IVS9+94G>A | c.1388+94G>A | - | Unknown | Uncertain significance |
| IVS9+141A>G | c.1388+141A>G | - | Unknown | Uncertain significance |
| IVS9-68T>C | c.1388-68T>C | - | Unknown | Uncertain significance |
| IVS9-36C>G | c.1389-36C>G | - | Unknown | Uncertain significance |
| IVS9-5T>A | c.1389-5T>A | - | Unknown | Uncertain significance |
| p.F426V | c.1393T>G | p.(Phe465Val) | Unknown | Uncertain significance |
| p.P428S | c.1399C>T | p.(Pro467Ser) | Unknown | Uncertain significance |
| p.E429G | c.1403A>G | p.(Glu468Gly) | Unknown | Uncertain significance |
| p.Q432Q | c.1413G>A | p.(Gln471Gln) | Unknown | Likely benign |
| p.K480Tfs*6 | c.1439_1445del7 | p.(Lys480ThrfsTer6) | Severe (Null) | Pathogenic |
| p.K441N | c.1440G>C | p.(Lys480Asn) | Unknown | Uncertain significance |
| p.D443N | c.1444G>A | p.(Asp482Asn) | Unknown | Likely benign |
| p.L444fs*0 | c.1447_1462del | p.(Leu483fsTer0) | Severe (Null) | Pathogenic |
| p.L444_M450delinsW | c.1447-1466delTGins | p.(Leu483_Met489delinsTrp) | Unknown | Uncertain significance |
| p.L444Rfs*5 | c.1448delT | p.(Leu483ArgfsTer5) | Severe (Null) | Pathogenic |
| p.L444P | c.1448T>C | p.(Leu483Pro) | Severe | Pathogenic |
| p.L444P + p.R(-23)G | c.1448T>C(;)49A>G | p.(Leu483Pro)(;)(Arg17Gly) | Severe;Unknown | Pathogenic;Uncertain significance |
| p.L444P / p.E326K | c.[1448T>C];[1093G>A] | p.[(Leu483Pro)];[(Glu365Lys)] | Severe/Risk Variant | Pathogenic/Benign |
| p.L444P + p.A456P | c.[1448T>C;1483G>C] | p.[(Leu483Pro;Ala495Pro)] | Severe + Unknown | Pathogenic + Uncertain significance |
| RecA456P | c.[1448T>C;1483G>C] | p.[(Leu483Pro;Ala495Pro)] | Severe | Pathogenic |
| RecNcil | c.[1448T>C;1483G>C;1497G>C] | p.[(Leu483Pro;Ala495Pro;Val499Val)] | Severe | Pathogenic |
| RecNcil / p.T369M | c.[1448T>C;1483G>C;1497G>C];[1223C>T] | p.[(Leu483Pro;Ala495Pro;Val499Val)];[(Thr408Met)] | Severe / Risk variant | Pathogenic/Uncertain significance |
| RecNcil + p.T369M | c.[1448T>C;1483G>C;1497G>C;1223C>T] | p.[(Leu483Pro;Ala495Pro;Val499Val;Thr408Met)] | Severe + Risk variant | Pathogenic + Uncertain significance |
| RecD, E, or AZRecTL (p.L444P, p.A456P, p.V460V, +92G>A) | c.[1448T>C;1483G>C;1497G>C,+92G>A] | NA | Severe | Pathogenic |
| p.L444P + RecG or p.L444P + Rec6b (p.L444P, +92G>A) | c.[1448T>C;+92G>A] | NA | Severe + Severe | Pathogenic + Pathogenic |
| p.L444R | c.1448T>G | p.(Leu483Arg) | Unknown | Likely pathogenic |
| p.D445Efs*4 | c.1452delC | p.(Asp484GlufsTer4) | Severe (Null) | Pathogenic |
| p.A446T | c.1453G>A | p.(Ala485Thr) | Unknown | Uncertain significance |
| p.A446A | c.1455A>G | p.(Ala485Ala) | Unknown | Likely benign |
| p.L449L | c.1464G>C | p.(Leu488Leu) | Unknown | Likely benign |
| p.M450Afs*15 | c.1465_1466del | p.(Met489AlafsTer15) | Severe (Null) | Pathogenic |
| p.H451R + p.G421D | c.[1469A>G;1379G>A] | p.[(His490Arg);(Gly460Asp)] | Unknown + Unknown | Uncertain significance + Uncertain significance |
| p.P452L | c.1472C>T | p.(Pro491Leu) | Unknown | Uncertain significance |
| p.P452P | c.1473C>T | p.(Pro491Pro) | Unknown | Likely benign |
| p.D453N | c.1474G>A | p.(Asp492Asn) | Unknown | Uncertain significance |
| p.D453L | c.[1475A>T;1474G>C] | p.(Asp492Leu) | Unknown | Uncertain significance |
| p.G454D | c.1478G>A | p.(Gly493Asp) | Unknown | Uncertain significance |
| p.A456P | c.1483G>C | p.(Ala495Pro) | Unknown | Uncertain significance |
| p.A456S | c.1483G>T | p.(Ala495Ser) | Unknown | Uncertain significance |
| p.V457D | c.1487T>A | p.(Val496Asp) | Unknown | Uncertain significance |
| p.V457A | c.1487T>C | p.(Val496Ala) | Unknown | Uncertain significance |
| p.V458L | c.1489G>(T or C) | p.(Val497Leu) | Unknown | Uncertain significance |
| p.V458E | c.1490T>A | p.(Val497Glu) | Unknown | Uncertain significance |
| p.V459V | c.1494C>T | p.(Val498Val) | Unknown | Likely benign |
| p.V460L | c.1495G>C | p.(Val499Leu) | unknown | Likely pathogenic |
| p.V460M | c.1495G>A | p.(Val499Met) | Unknown | Likely pathogenic |
| p.V460V | c.1497G>C | p.(Val499Val) | Unknown | Likely benign |
| p.L461P | c.1499T>C | p.(Leu500Pro) | Unknown | Uncertain significance |
| p.L461P + IVS10+1G>T | c.[1499T>C;1505+1G>T] | p.(Leu500Pro) | Unknown + Severe | Uncertain significance + Pathogenic |
| p.R463C | c.1504C>T | p.(Arg502Cys) | Severe | Pathogenic |
| p.R463S | c.1504C>A | p.(Arg502Ser) | Unknown | Pathogenic |
| p.R463P | c.1505G>C | p.(Arg502Pro) | Unknown | Uncertain significance |
| p.R463H | c.1505G>A | p.(Arg502His) | Unknown | Pathogenic |
| p.R463Qfs*2 + IVS10-1G>A | c.[1505G>A;1506-1G>A] | p.(Arg502GlnfsTer2) | Severe (Null) | Pathogenic |
| IVS10+1G>T | c.1505+1G>T | - | Severe | Pathogenic |
| IVS10+3G>A | c.1505+3G>A | - | Unknown | Uncertain significance |
| IVS10+8C>A | c.1505+8C>A | - | Unknown | Uncertain significance |
| IVS10+49G>A | c.1505+49G>A | - | Unknown | Uncertain significance |
| IVS10-10T>G | c.1506-10T>G | - | Unknown | Uncertain significance |
| IVS10-4C>T | c.1506-4C>T | - | Unknown | Uncertain significance |
| p.S464P | c.1507T>C | p.(Ser503Pro) | Unknown | Uncertain significance |
| p.S465P | c.1510T>C | p.(Ser504Pro) | Unknown | Uncertain significance |
| p.K466K | c.1515G>A | p.(Lys505Lys) | Unknown | Likely benign |
| p.G478R | c.1549G>C | p.(Gly517Arg) | Unknown | Likely pathogenic |
| p.E481Xfs | ? | p.(Glu520Terfs) | Severe (Null) | Pathogenic |
| p.T482K | c.1562C>A | p.(Thr521Lys) | Unknown | Uncertain significance |
| p.S484L | c.1568C>T | p.(Ser523Leu) | Unknown | Uncertain significance |
| p.S488T | c.1579T>A | p.(Ser527Thr) | Unknown | Uncertain significance |
| p.I489V | c.1582A>G | p.(Ile528Val) | Unknown | Uncertain significance |
| p.H490Y | c.1585C>T | p.(His529Tyr) | Unknown | Uncertain significance |
| p.H490R | c.1586A>G | p.(His529Arg) | Unknown | Uncertain significance |
| p.R495H | c.1601G>A | p.(Arg534His) | Unknown | Uncertain significance |
| p.R496H | c.1604G>A | p.(Arg535His) | Mild | Pathogenic |
| p.Q497* | c.1606C>T | p.(Gln536Ter) | Severe (Null) | Pathogenic |
| p.Q497R | c.1607A>G | p.(Gln536Arg) | Unknown | Uncertain significance |

Note: This table was organized from “Classification of GBA1 Variants in Parkinson’s Disease: The GBA1-PD Browser, PMID: 36598340”.
